# A novel endogenous selection marker for the diatom *Phaeodactylum tricornutum* based on a unique mutation in phytoene desaturase 1

**DOI:** 10.1038/s41598-019-44710-5

**Published:** 2019-06-03

**Authors:** Yogesh Taparia, Aliza Zarka, Stefan Leu, Raz Zarivach, Sammy Boussiba, Inna Khozin-Goldberg

**Affiliations:** 1 Microalgal Biotechnology Laboratory, French Associates Institute for Agriculture & Biotechnology of Drylands, The Jacob Blaustein Institutes for Desert Research, Ben-Gurion University of the Negev, Sede-Boqer Campus, Midreshet Ben-Gurion, 8499000 Israel; 2 Department of Life Sciences, Faculty of Natural Sciences, Ben-Gurion University of the Negev, Beer-Sheva, 8410501 Israel

**Keywords:** Molecular engineering in plants, Expression systems

## Abstract

*Phaeodactylum tricornutum* is a well-developed model diatom for both marine ecology and microalgal biotechnology, which has been enabled by the sequenced genome and the availability of gene delivery tools, such as biolistic transformation and *E*. *coli*-mediated conjugation. Till now, these tools have mainly relied on two selectable markers of bacterial origin which confer resistance to antibiotics Zeocin and nourseothricin. An alternative cost-effective and preferably endogenous selectable marker would facilitate gene stacking efforts through successive transformation or conjugation. We performed UV-mutagenesis of *P*. *tricornutum* to obtain mutations in the phytoene desaturase (PDS) gene, conferring resistance to the bleaching herbicide norflurazon. Two mutants displaying high tolerance to norflurazon and carrying unique mutations in PtPDS1 (PHATRDRAFT_45735) were selected. These mutants revealed novel point mutations at a conserved residue Gly290 to Ser/Arg. Homology-based structural modeling of mutated PDS1, over a resolved crystallographic model of rice PDS1 complexed with norflurazon, suggests steric hindrance by bulkier residue substitution may confer herbicide resistance. We report the characterization of PtPDS1 mutants and the development of the first endogenous selectable marker in diatoms suitable for industrial strain development, with the added benefit of biocontainment. The plasmid carrying the mutated PDS1 as a selection marker and eGFP as a reporter was created. An optimized biolistic transformation system is reported which allowed the isolation of positive transgenic events at the rate of 96.7%. Additionally, the ease of *in vivo* UV-mutagenesis may be employed as a strategy to create PDS-norflurazon-based selectable markers for other diatoms.

## Introduction

Diatoms are an extremely morphologically diverse group of photosynthetic microalgae that inhabit nearly all aquatic environments and contribute approximately 20 to 40% of atmospheric O_2_^1,2^. In marine environments, photosynthetic diatoms display rapid growth by assimilating available nutrients, resulting in blooms. The capability of diatoms to use photosynthetic energy for rapid growth makes them excellent target organisms for microalgal biotechnology and genetic engineering^3–5^. As many diatoms are oleaginous species that are able to accumulate high amounts of storage lipids, they have attracted significant attention as potent biofuel organisms.

The pennate oleaginous diatom *Phaeodactylum tricornutum* has been engineered for enhanced fatty acid biosynthesis^6,7^ and storage lipid accumulation^8–10^ and improved LC-PUFA production^11–13^. This diatom is an emerging synthetic biology chassis, enabling synthesis of a variety of compounds, and a proven host for recombinant protein production. The production of triterpenoid compounds^14^, reconstruction of vanillin biosynthesis^15^, therapeutic peptide production^16–18^, and trophic conversion^19,20^ have been achieved in this industrially relevant diatom.

Numerous genetic engineering tools, such as gene delivery^21–24^, genome editing^15,25–29^ and gene silencing^30^, have been established to explore the genome information and empower the biotechnological exploitation of *P*. *tricornutum*. These tools depend on an efficient selectable marker for the selection of transgenic events. Limited availability of efficient and cost-effective selection schemes for nuclear transformation in *P*. *tricornutum* hinders the prospect of simultaneous or tandem delivery of multiple traits for gene stacking or engineering complex biosynthetic pathways. Furthermore, *P*. *tricornutum* was shown to be insensitive to a wide range of commonly used antibiotics and herbicides^31^. Currently, only two selectable markers *sh-ble* and *nat* are predominantly used to select transgenic events in *P*. *tricornutum*. Recently another selectable marker, blasticidin-S deaminase^32^ has been reported for *P*. *tricornutum*. However, these selectable markers are of bacterial origin and not suitable for generation of transgenic events for commercial use and are likely to face regulatory hurdles. Disruption of native genes leading to loss of function, such as PtUMPS and PtAPT, have been reported as counter selectable markers in *P*. *tricornutum* for genome editing applications^29^. However, their utility in generating transgenic events is limited due to the availability of a functional copy in the recipient cells which metabolizes the synthetic metabolite analog into a suicidal product leading to mortality of all cells. On the other hand, gain of function mutations in native genes may be used as endogenous selectable markers. Such endogenous selectable markers based on herbicide resistance offer an efficient and cost effective alternative to selectable markers of bacterial origin.

Herbicides, coupled with an endogenous or transgenic source of genetic resistance, have been routinely deployed as selectable markers for nuclear or plastid transformation in higher plants. The bleaching herbicide norflurazon competitively binds phytoene desaturase (PDS), the first enzyme in the carotenoid biosynthesis pathway, which catalyzes the introduction of two double bonds into phytoene, yielding ζ-carotene via the intermediate carotenoid phytofluene^33^. Several mutations in the PDS coding sequence conferring resistance to norflurazon have been reported^34,35^ and developed as selectable markers in higher plants and green microalgae, but none in microalgae with high biotechnological potential, which belong to Stramenopiles, such as diatoms and eustigmatophytes (e.g., *Nannochloropsis* spp.).

Here, we report the *in vivo* UV-mutagenesis of the native phytoene desaturase-1 (PHATRDRAFT_45735; *P*. *tricornutum* JGI database v2.0) coding sequence of the *P*. *tricornutum* strain UTEX 646 and the isolation of novel mutants conferring resistance to norflurazon. The PtPDS1 sequence from the most tolerant mutant was used to create the first endogenous selectable marker in *P*. *tricornutum* that is suitable for efficient selection of transgenic events and use in the production of industrial strains ensuring biocontainment.

## Results

### Determination of an optimal norflurazon selection concentration

**Figure 1:**
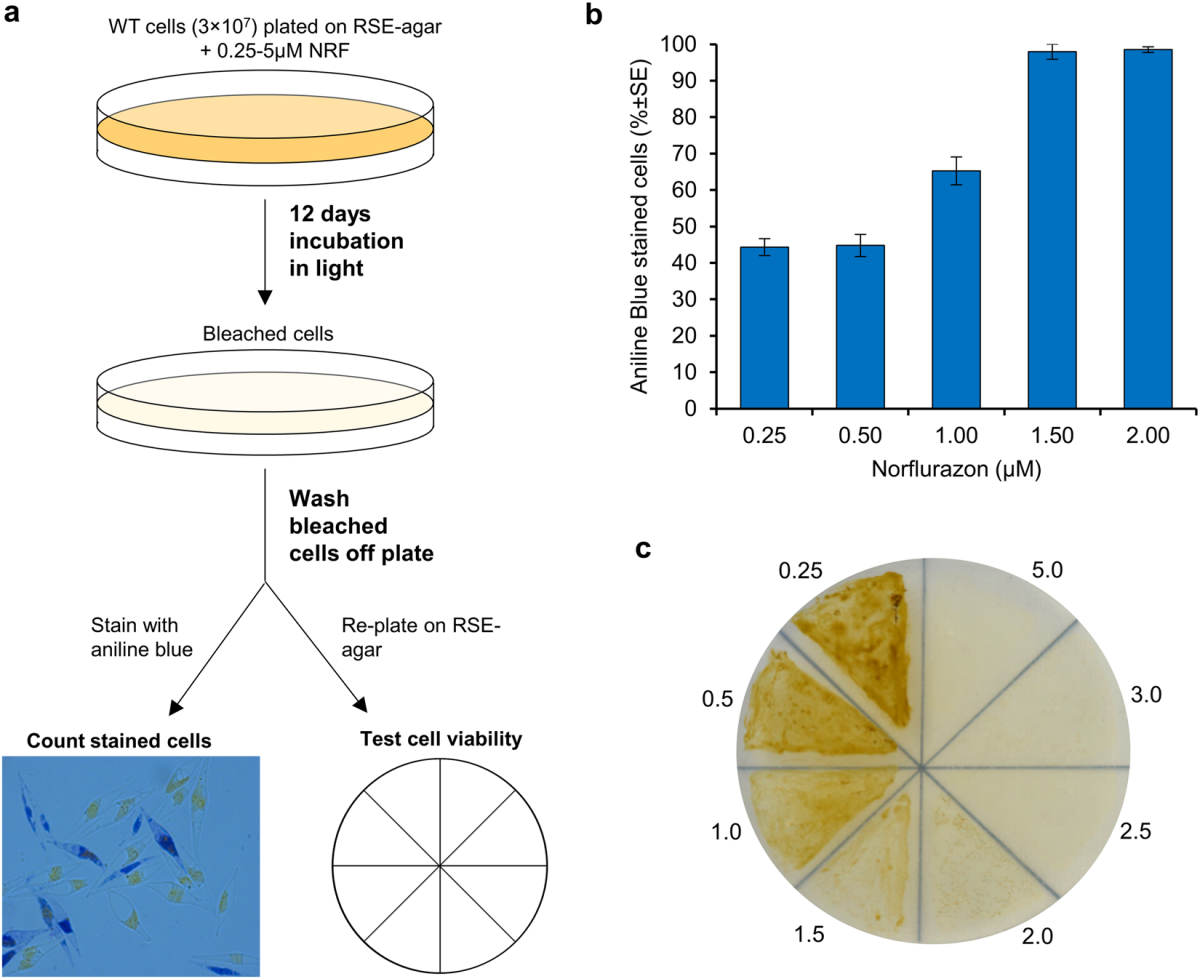
Determination of sensitivity of WT *P*. *tricornutum* UTEX 646 to norflurazon. (**a**) Schematic representation of experimental procedure to determine norflurazon sensitivity in WT *P*. *tricornutum* cells. 3 × 10^7^ WT cells were plated on RSE-agar supplemented with norflurazon concentrations ranging from 0.25–5 µM. Cells were washed off plates after 12 days of incubation and stained with Aniline Blue for microscopic evaluation. (**b**) Percent Aniline Blue stained cells averaged over several non-overlapping fields. Error bars represent standard error. (**c**) Non-selective recovery of bleached WT cells to determine an optimal norflurazon selection concentration. Washed off RSE-agar + norflurazon cells were re-plated onto RSE-agar and grown under illumination of 60 μmol m^−2^ s^−1^ for 15 days to allow the recovery of any viable cells post-herbicide treatment. Numerical figures indicate treatment concentration of norflurazon before re-plating.

For the development of an endogenous selection marker, suitable for genetic engineering in *P*. *tricornutum*, we focused on the resistance to the herbicide norflurazon. The sensitivity of WT *P*. *tricornutum* to increasing concentrations of norflurazon (0.25–5 μM) was tested by plating cells on RSSE-agar and incubation for 12 days followed by microscopy of Aniline Blue stained cells to assess cell damage as indicated by the cellular permeabilization of the stain (Fig. 1a,b). Uniform Aniline Blue permeabilization and staining of treated WT *P*. *tricornutum* cells was observed at 1.5 µM and higher norflurazon concentrations, which was associated with a breakdown of cellular membranes, a desintegration of the chloroplast and a loss of cell viability (Supplementary Fig. S1). Norflurazon-treated cells that were re-plated for recovery on the RSSE-agar medium free of norflurazon displayed 100% mortality at 2.5 µM and higher herbicide concentrations after 15 days of incubation (Fig. 1c). Based on these observations, selection was tested at 3 and 5 µM norflurazon concentrations in biolistic transformation experiments.

### Isolation of *P. tricornutum* norflurazon-resistant PtPDS1 mutants and their characterization

Based on the results above and a preliminary assay of WT *P*. *tricornutum* sensitivity to norflurazon in liquid media (not shown), we chose 15 µM norflurazon concentration to enable the selection of highly resistant mutants and to limit escapes. Accordingly, UV-mutagenized colonies, obtained after exposure to different durations of UVB (see materials and methods), were selected on solid media containing 15 µM norflurazon. After 15 days, the two fastest growing norflurazon-resistant mutants (M1 and M2) were recovered from a 2-min UVB exposure.

**Figure 2:**
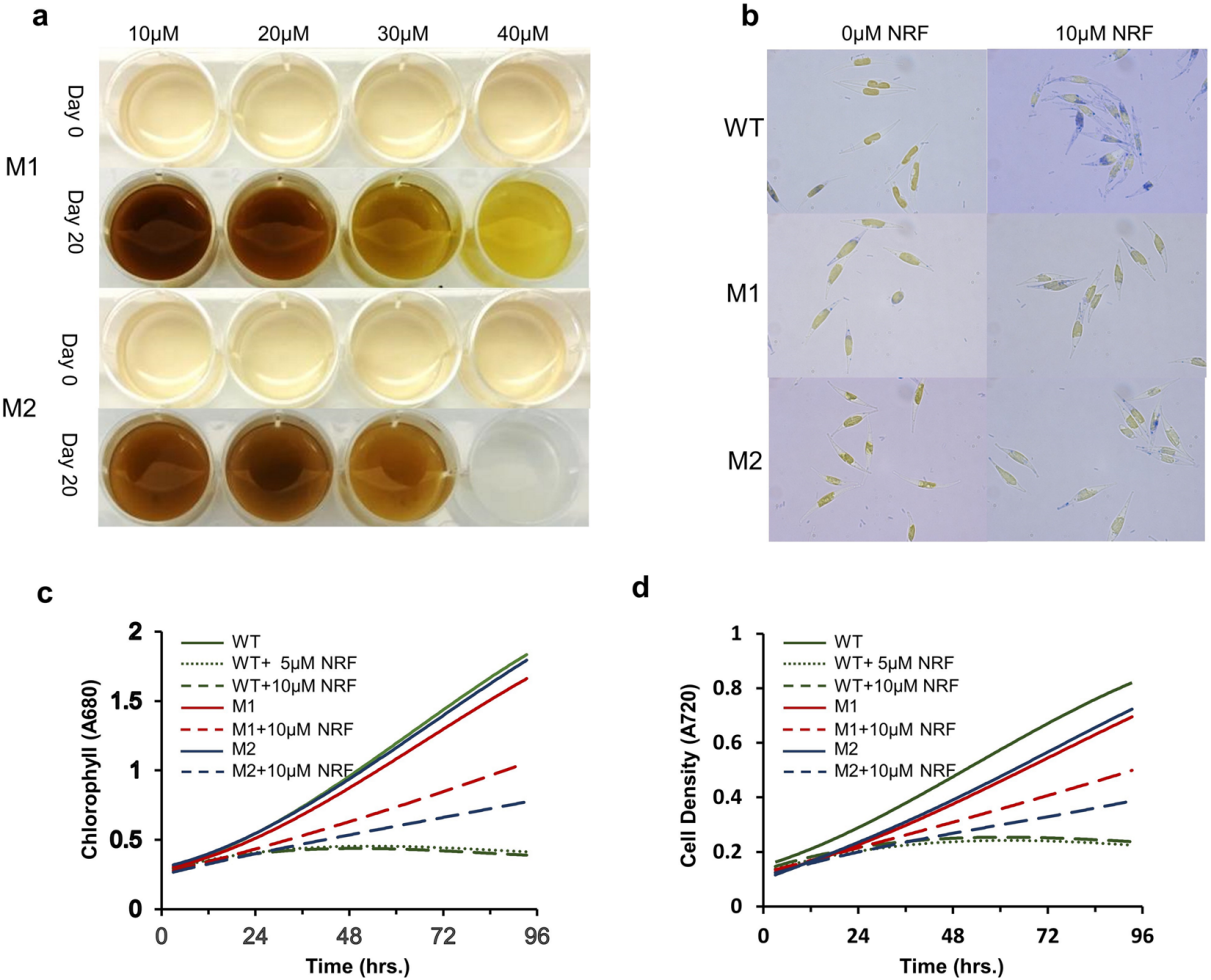
Tolerance of *P*. *tricornutum* mutants M1 and M2 to norflurazon. (**a**) Mutants M1 and M2 cultures in mid-log phase were diluted to 5 × 10^5^ cells per mL and treated with norflurazon (10–40 µM) and cultured under illumination of 75 μmol m^−2^ s^−1^ for 20 days. Mutant M1 displayed greater tolerance to norflurazon. (**b**) WT and mutants M1 and M2 were growth in liquid cultures in a multi-cultivator for 93 h and examined for cell viability with Aniline Blue staining at the end of the experiment. (**c**,**d**) Growth characterization of mutants M1 and M2 in comparison with WT in the presence or absence of norflurazon (NRF).

A preliminary characterization of the M1 and M2 mutants was done in a 12-well plate, where mutants were exposed to a range of norflurazon concentrations between 10 and 40 µM. At the highest concentration of 40 µM norflurazon, M1 displayed chlorosis, whereas M2 was completely bleached at the end of day 20 of incubation (Fig. 2a). Both mutants displayed tolerance to norflurazon concentrations lower than 30 μM. Next, we compared the growth of mutants and WT in a column photobioreactor, with and without 5 and 10 µM norflurazon. Mid-log phase cultures were diluted to chlorophyll 2 mg L^−1^ at the start of experiment; chlorophyll and cell density were estimated by recording A680 and A720, respectively. By the end of experimental period, norflurazon-treated WT cells displayed greater Aniline Blue staining than their treated M1 and M2 counterparts (Fig. 2b), revealing greater WT sensitivity to herbicide and tolerance in the mutant lines. In the presence of 10 µM norflurazon, M1 and M2 mutants displayed a specific growth rate of 0.211 and 0.141 Abs-680 day^−1^, respectively, whereas chlorophyll production in WT cultures was completely arrested at 48 h, in the presence of 5 μM norflurazon (Fig. 2c). Untreated WT displayed slightly better chlorophyll accumulation than the M1 or M2 cultures in each experimental repetition. A similar trend was observed for cell density (A720) in norflurazon-treated and untreated cultures (Fig. 2d).

### Phylogenetic and sequence analysis of PtPDS1 mutants

The plastid-targeted *P*. *tricornutum* PDS1 displayed the highest homology to P of other diatoms (Fig. S2). Within the phylogenetic tree for chloroplastic phytoene desaturases, *P*. *tricornutum* PDS1 clustered more closely to early divergent green microalgae (Prasynophytes), which form a clade separate from higher green algae (Chlorophytes) and land plants. Brown algal PDS peptide sequences were the most divergent and were used to root the phylogenetic tree.

Sanger sequencing of the cloned PtPDS1 CDS from mutant strains and the alignment of its translated peptide sequence to WT PtPDS1 showed substitutions at position Met28Ile (M1), Gly290Ala/Ser (M1/M2) (Supplementary Fig. S3a) and Arg494Cys (WT) of the amino acid sequence. Of these three substitutions, Gly290 was the only conserved residue (Supplementary Fig. S3b). Moreover, the Met28Ile substitution occurred in the chloroplast transit peptide (N1-MMFHYKTGSSWFLLLSASITTTLTTTTMTTTHAFAP-C36), which is predicted to be cleaved within the ASAFAP motif (Phe34)^36–38^. Mapping of known conserved residue substitutions conferring norflurazon resistance to the PtPDS1 peptide sequence confirms that Gly290Ala/Ser is a novel mutation (Fig. S3b).

**Figure 3:**
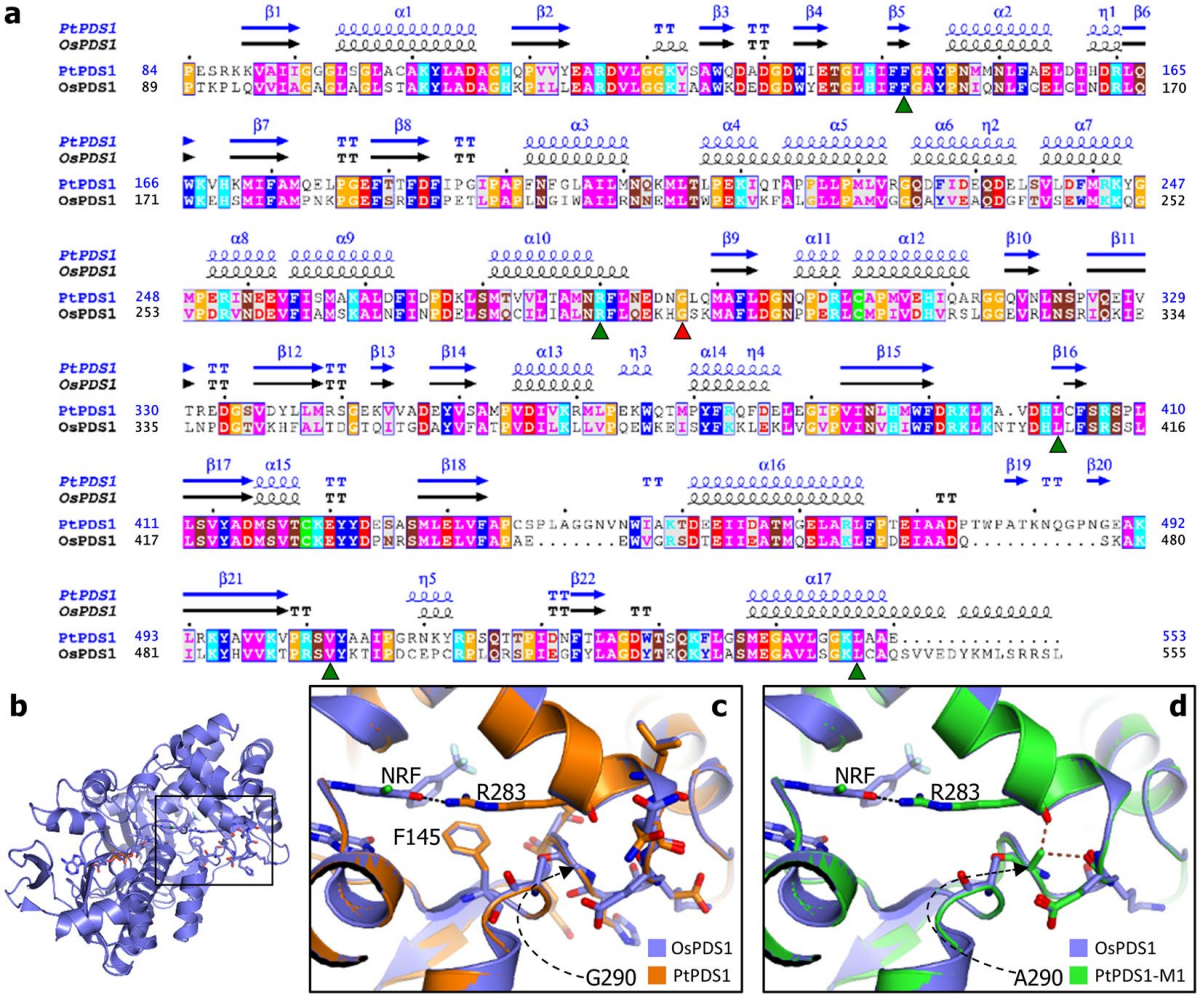
*In-silico* analysis of PtPDS1-M1 and M2 mutant sequences deduces a basis for norflurazon tolerance. (**a**) Representation of secondary structure alignments between PtPDS1 (target; SWISS-MODEL) and OsPDS1 (template; PDB: 5mogA) shows 59.51% sequence identity and sequence similarity score of 0.48. Sites of previously reported norflurazon conferring substitutions (green triangles) and PtPDS1-M1/M2 (red triangle) are indicated. (**b**) Representation of OsPDS1 (PDB: 5mogA) resolved structure complexed with Norflurazon and FAD. (**c**) Enlarged inset (shown in **d**) overlaid with modeled WT PtPDS1 displays structural homology at norflurazon biding site. (**d**) R283-Norflurazon (NRF) hydrogen bond (black dotted line) may be disrupted by Gly290Ala (M1) residue change due to side group clashes (brown dotted lines) leading to reduced affinity of PtPDS-M1 for norflurazon observed as norflurazon tolerance in M1.

Alignment of the PtPDS1 and OsPDS1 (GenBank accession number AF049356) peptide sequences showed a 59.51% sequence identity and a sequence coverage of 76%. Alignment between secondary structures of PtPDS1 and OsPDS1 showed highly similar topologies (Fig. 3a). The homology-based modeling of PtPDS1 over the OsPDS1 crystallographic structure (PDB: 5MOG.A, Fig. 3b) had a QMEAN value of −1.98 indicating high structural similarity and high accuracy of the model. Phe145 and Arg283 (Fig. 3c) residues are known to interact with norflurazon and allow its stable binding and inhibition of the phytoene desaturase enzyme. The PtPDS1 modeled structure overlaid on norflurazon-inhibited OsPDS1 indicated a clash between Gly290Ala (M1) and the backbone carbonyls of Arg283 and Glu287, which may interfere with residues stabilizing norflurazon in WT phytoene desaturase-1 enzyme (Fig. 3c), which may explain the basis of norflurazon resistance in mutants.

### Optimization of *P. tricornutum* transformation with the pPtPDS1-M1 construct

**Figure 4:**
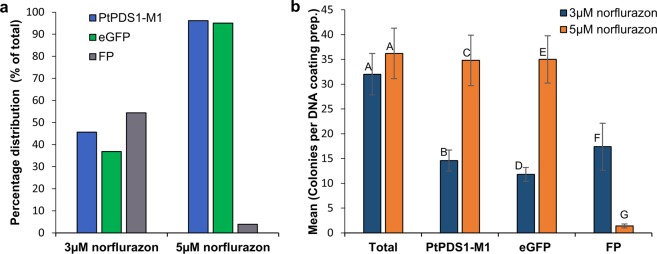
Optimization of selection efficiency with 3 or 5 µM norflurazon by biolistic transformation of pPtPDS1-M1 plasmid. Five microcarrier-DNA coating preparations were shot for each selection concentration of norflurazon. All putative transformants that appeared by day 12 of selection were screened by PCR for amplification of eGFP or PtPDS1-M1. (**a**) Percentage distribution out of total number colonies screened: 160 (3 µM norflurazon) and 181 (5 µM norflurazon). (**b**) Average number of colonies per microprojectile-DNA coating preparation of colonies screened by PCR. Bars marked with same alphabets indicate non-significant difference at α = 0.05. Error bars depict standard error values. FP = False positive.

Next, we used the PDS1 sequence from mutant M1 to construct a pPtPDS1-M1vector (Fig. S4), harboring eGFP as a reporter (for details, please refer to Materials and Methods). An efficient norflurazon concentration, suitable for transformant selection, would maximize the selection of transgenic events and yet minimize the occurence of  false positives. Transformation efficiency and the rate of false positive events were compared at 3 and 5 µM norflurazon selection post-biolistic delivery of pPtPDS1-M1 expression vector (Fig. 4).

A total of 160 (3 µM norflurazon) and 181 (5 µM norflurazon) putative transgenic events were PCR screened for the presence of eGFP or PtPDS1-M1 (Fig. 4a,b). The mean number of colonies per microcarrier coating reaction in each selection regime were about 32–36 and statistically non-significant (p = 0.54) (Fig. 4b). However, selection at 5 µM norflurazon produced significantly more Pt-PDS1 positive lines (p = 0.003) than 3 µM norflurazon: 96.1% vs. 45.6%, respectively (Fig. 4a). A similar trend was seen for eGFP positive lines: 96.7% vs. 36.9% (p = 0.002). Selection at 5 µM norflurazon effectively reduced non-transgenic escapes to ~4%, compared to 50% of the total colonies screened at 3 µM (p = 0.014) (Fig. 4a,b).

### Validation of transgene integration and expression

**Figure 5:**
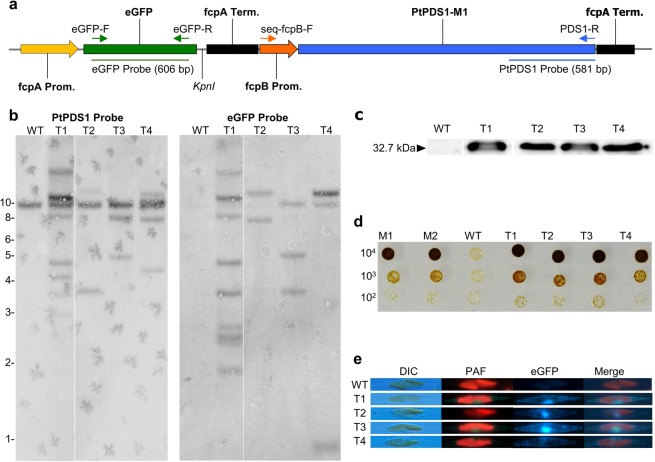
Characterization of transgenic lines. (**a**) Schematic representation of pPtPDS1-M1 vector depicting PCR primer binding sites and restriction sites used for validation of transgene insertion in to *P*. *tricornutum* nuclear genome. (**b**) Southern blot of genomic DNA digested with *KpnI* restriction enzyme was hybridized with a *PDS1* probe (581 bp), stripped and re-hybridzed with *eGFP* fragment (606 bp) to confirm transgene integration in nuclear genome of *P*. *tricornutum*. (**c**) Western blot of crude protein extracts from transgenic events was probed with anti-GFP antibody to confirm eGFP expression. (**d**) Norflurazon tolerance assay of transgenic events on RSE-agar + 5 µM Norflurazon to confirm expression of PtPDS-M1 from integrated cisgene. (**e**) Cytoplasmic eGFP expression was confirmed in transgenic lines through fluorescent microscopy (see Materials and Methods). DIC - differential interference contrast; eGFP -enhanced green fluorescent protein; PAF - plastidial auto-fluorescence.

Putative transgenic events screened by PCR, for the integration of PtPDS1-M1 and eGFP (Supplementary Fig. S5) in the nuclear genome of the transformants T1, T2, T3, and T4, displayed positive amplification. Furthermore, Southern blotting of *KpnI*-digested genomic DNA of transgenic events and WT with eGFP and PtPDS1 probes, indicated in Fig. 5a, validated transgene integration (Fig. 5b). Cytoplasmic eGFP protein corresponding to 37.2 kDa was detected in a western blot of transgenic events (Fig. 5c). *In vivo* expression of cytoplasmic eGFP was observed by fluorescent microscopy in transgenic lines (Fig. 5e). Spotted 10^4^–10^2^ cells of transgenic lines on RSE-agar + 5 µM norflurazon plates displayed no growth inhibition, confirming PtPDS1-M1 expression (Fig. 5d).

### Evaluation of long-term expression stability of transgenic lines

**Figure 6:**
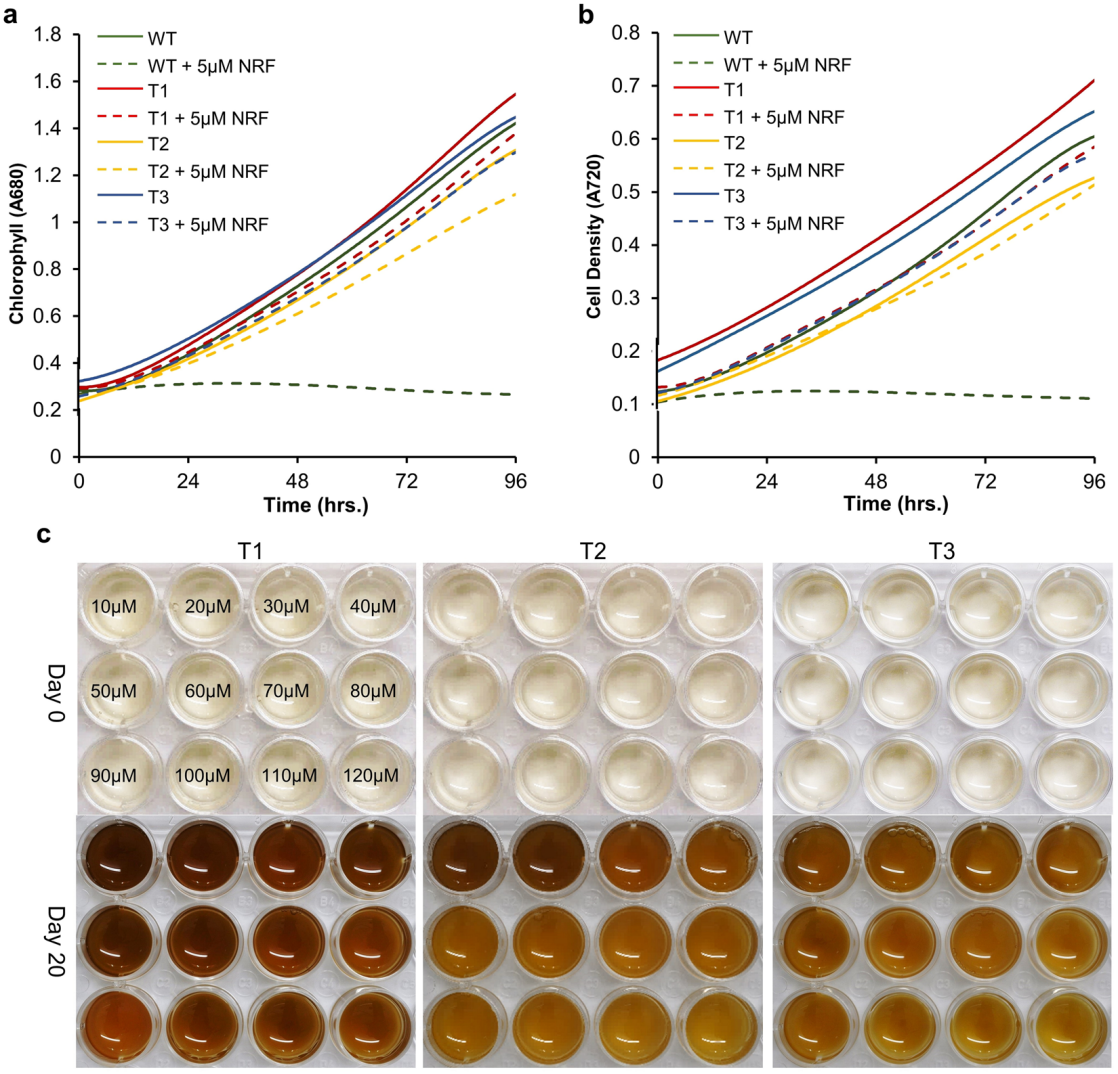
Evaluation of long-term expression stability of transgenic lines. Transgenic lines T1, T2 and T3 (from Fig. 5b), displaying different transgene copy number and maintained in nonselective medium for nearly 48 months were evaluated for stable expression of selectable marker. (**a**,**b**) Growth characterization of transgenic lines T1, T2 and T3 compared with WT in the presence or absence of 5 µM norflurazon (NRF). (**c**) Transgenic lines T1, T2 and T3 grown to mid-log phase were diluted to 5 × 10^5^ cells per mL and treated with norflurazon (10–120 µM) and cultured under illumination of 75 μmol m^−2^ s^−1^ for 20 days.

Transgenic lines (T1, T2 and T3), maintained without selection for about 48 months, representing a range of transgene copy number integration were characterized for norflurazon sensitivity and eGFP expression. Transgenic lines cultured in column photobioreactor, with or without 5 µM norflurazon displayed robust growth over three repetitions of the experiment (Fig. 6a,b). In the presence of 5 µM norflurazon, transgenic lines displayed minimal inhibition of growth when compared to untreated controls, including wild-type. Furthermore, transgenic lines displayed tolerance over a range of norflurazon concentrations (10–120 µM) and remained uninhibited in a plate-based assay of norflurazon sensitivity (Fig. 6c). Integration of *eGFP* and PtPDS1-M1 was validated by PCR (Supplementary Fig. S5a). Stable eGFP expression in all three transgenic lines was validated by fluorescence microscopy (Supplementary Fig. S6b).

## Discussion

The diatom *P*. *tricornutum* has developed into the primary model microalga for biotechnological exploitation, specifically for lipids, LC-PUFA and recombinant protein production, and a wide range of molecular tools used in developing this diatom into a valuable synthetic biology chassis have been reported^15,39^. UV-mutagenesis is a simple method for the introduction of dominant genic mutations in a wide range of microorganisms. In *P*. *tricornutum*, UV-mutagenesis has been successfully employed to enhance EPA content up to 44%^40^. Here, we report a novel dominant endogenous selection marker in diatoms for the transformation of *P*. *tricornutum*, based on a novel amino acid substitution in PtPDS1 (PHATRDRAFT_45735; *P*. *tricornutum* JGI database v2.0) conferring resistance to the bleaching herbicide norflurazon.

Through random or targeted mutagenesis of the PDS enzyme, norflurazon resistance has been successfully developed as an endogenous selectable marker in several algae^41–47^ and plant species^48,49^. With respect to the PtPDS1 sequence, five amino acid substitutions at highly conserved residues in the PDS enzyme that are known to confer resistance to norflurazon have been reported so far: Phe145Val^43^, Arg283Pro/Ser/Thr/Cys^49–52^, Leu403Pro^34^, Val505Gly^34,53,54^, and Leu550Arg/Phe^41,42,44–47^. These residue substitutions were all shown to occur in the vicinity of the reaction center comprising the FAD-binding site and the plastoquinone/norflurazon binding pocket in the Rice PDS (OsPDS1) crystal structure^33,55^. *In vivo* UV mutagenesis and stringent selection allowed isolation of norflurazon-resistant mutants of *P*. *tricurnutum* with novel substitutions at a highly conserved residue, Gly290. Homology modeling of the PtPDS1 peptide over the OsPDS1-elucidated structure, suggests that Gly290Ala/Ser substitutions introduce bulky residue that clash with the backbone carbonyls of Arg283 and Glu287, which may destabilize norflurazon interaction with phytoene desaturase enzyme and likely result in norflurazon resistance.

Nuclear transformation in *P*. *tricornutum* is routinely achieved by two selectable markers of bacterial origin, *Sh-ble*- Zeocin™ and *nat*- nourseothricin. Apart from *Sh-ble* and *nat*, *nptII*-geneticin and *cat*-chloramphenicol are the only other selectable markers that have been used with less success in transformation of *P*. *tricornutum* and therefore not used routinely. Use of alternate selectable markers such as *nptII* and *nat* are fraught with difficulties, such as being effective only at higher concentrations and at reduced salt strength in the selection medium. Moreover, an array of commonly used antibiotic selection agents, such as kanamycin, streptomycin, spectinomycin, and hygromycin are ineffective as selection agents in *P*. *tricornutum*^22,56^. Commercial herbicides and their corresponding resistance genes provide a cost-effective and efficient selection scheme when genetically encoded antibiotic resistance markers are unavailable. However, widely used herbicides, such as glyphosate, bialaphos, phosphinothricin, sulfometuron-methyl, chlorosulfuron, and imazapyr, have proven to be ineffective in killing *P*. *tricornutum*^22^. Due to this dearth of efficient selectable markers, PDS-norflurazon resistance based selection system was explored in *P*. *tricornutum*. In contrast to selectable markers of bacterial origin, endogenous PDS1 gene, resistant to norflurazon provides additional benefits of efficient selection, biocontainment and cost-effectiveness. When compared on a cost per liter basis for producing one liter of selective medium, norflurazon, Zeocin™ and nourseothricin, respectively, are ca. USD 2 (Merck, USA), 20 (InVivoGen, San Diego, USA) and 3000 (Merck, USA).

In order to develop PtPDS1-M1 into an efficient selectable marker, optimization of the selection pressure on biolistic-transformed cells was carried out at 3 and 5 µM norflurazon concentrations. Selection at 5 µM norflurazon allowed significant improvement in selection of positive transgenic lines at the rate of 96.7% of total colonies obtained. Similarly, non-transgenic escapes were limited to 3.8% of the total colonies screened. With 5 µM norflurazon selection, on an average each microprojectile coating preparation produced 35 transformants as compared to ~100 with *sh ble-*Zeocin™; this may be attributed to the PtPDS1-M1 CDS length of 1875bp, which is five times that of *sh-ble* CDS.

The *Phaeodactylum tricornutum* genome contains two homologs of the phytoene desaturase enzyme viz. PtPDS1 (PHATRDRAFT_45735) and PtPDS2 (PHATRDRAFT_55102), of which only the former is functional^57^. A phylogenetic analysis of the PtPDS1 peptide sequence revealed close similarity to other diatom PDS sequences, which is in agreement with Dambek *et al*.^57^. Based on the endosymbiotic history of diatom chloroplasts, it would be expected that plastid-targeted nuclear-encoded proteins would share greater homology with their red algal endosymbiont ancestor; however, a recent study of the genes involved in the carotenoid biosynthesis of the chromist algae revealed that about two-thirds of these genes are closely related to the prasinophytes^58^. Retention of sequences from a prasinophyte source has been implicated in the evolution of fitness in diatoms and their capability to dominate the marine environment^59^. PtPDS1 clustered to a clade comprising diatoms, cryptomonads, and prasinophytes, which displayed divergence from the clade comprising the green algae, and the lower and higher plants, in agreement with Frommolt *et al*.^58^.

Norflurazon-resistant PDS mutants with instances of significant impairment of *in vivo* carotenogenic activity upon PDS mutagenesis have been reported^34,53,54^. *In vitro* characterization of mutations Leu550Phe/Arg and Phe145Val in rice PDS, under normal conditions, showed less than 5% activity compared to WT rice PDS except for Arg283Ser, which retained about 15% activity^55^. It has been proposed that such mutants may compensate for reduced carotenogenic activity by transcriptional up-regulation via retrograde signaling to the nucleus. On the other hand, norflurazon-resistant mutants without major retardation of carotene biosynthetic capability have been isolated^51^. Such mutants are preferable for exploitation as selectable markers as they would neither significantly alter cellular transcription nor adversely affect transgene expression. The two norflurazon-mutants, M1 and M2, displayed growth rates, comparable to WT under untreated conditions. The specific growth rates of WT, M1 and M2 lines displayed significant retardation in the presence of a killing concentration of norflurazon (10 µM), to 0%, 65% and 36%, respectively, when compared to their untreated controls.

Consequently, we used the PDS1 sequence from mutant M1 to develop a novel selectable marker for *P*. *tricornutum*. The established transformation vector pPha-T1^21^ expressing eGFP was modified by replacement of the *ShBle* selectable marker with PtPDS-M1 to create a test vector for nuclear transformation, pPtPDS1-M1 (Fig. 4a). After confirmation of successful transformation, a new transformation vector, pBS-PtPDS1-M1 (Fig. 4b), was created by ligation of an excised PtPDS1-M1 selectable marker cassette into the *SpeI* restriction site of the pBlueScript SK II (+) vector with extensive multiple cloning sites for insertion of additional transcriptional units.

Stable integration and expression of transgenes is essential for a transformation system to be considered reliable. To this end, three transgenic lines maintained over 48 months without selection were subjected to norflurazon sensitivity assay in plate and growth characterization in column photobioreactor. These transformants displayed enhanced tolerance range with slight inhibition at 120 µM norflurazon which is nearly three times the upper limit for mutant M1. Similar results were observed in the column photobioreactor, with minimal growth inhibition for transgenic lines in the presence of the herbicide. These results indicate that the PtPDS1-Gly290Ala is a functional enzyme, which displays resistance to norflurazon.

Identification of a novel endogenous dominant selection marker for the model species *P*. *tricornutum* is of great significance towards advancing synthetic biology approaches in this versatile and extensively researched species. Availability of an endogenous selection marker will not only facilitate the selection of successful transformants but will also allow the creation of cisgenic *P*. *tricornutum* events provided that only native sequences are repurposed for genetic engineering, so that regulatory hurdles for the cultivation and marketing of such products may be overcome.

## Conclusion

We demonstrate a simple *in-vivo* UV-mutagenesis strategy to isolate dominant mutants, *viz*. norflurazon resistance in the model diatom *P*. *tricornutum*. These novel mutants were characterized for herbicide resistive strength, and the coding sequence of PtPDS1 from M1 mutant with greater resistance was used to create the first successful endogenous selectable marker in *P*. *tricornutum* which is cost-effective, efficient and free of biocontainment concerns. In the future, domestication of type-IIS restriction sites within the selectable marker would make it a preferred selectable marker for use in large-scale transcriptional assembly methods such as Golden braid^60^ or Mobius assembly^61^ for engineering synthetic pathways in *P*. *tricornutum*.

## Materials and Methods

### Algal strain and culture conditions

An axenic culture of *P*. *tricornutum* UTEX 646 was maintained on Red Sea Salt Enriched (RSE)-agar (Red Sea, Israel)^62^ plates under illumination of 50 μmol photons m^−2^ s^−1^, at 22 °C. Liquid cultures (100 mL culture in a 250-mL Erlenmeyer flask) were routinely diluted and maintained in an exponential growth phase in an incubator shaker at 18 °C and 120 rpm, with the continuous illumination of 75 μmol photons m^−2^ s^−1^ under a CO_2_-enriched atmosphere (200 mL min^−1^).

### Determination of an effective norflurazon concentration for selection on solid medium

Agar plates, containing half-strength RSE salt supplemented with different concentrations of norflurazon (Millipore-Sigma, West Chester, USA) dissolved in DMSO (0.25, 0.5, 1.0, 1.5, 2.0, 2.5, 3.0 and 5.0 μM), were plated with 3 × 10^7^ cells from a mid-log phase wild type (WT) *P*. *tricornutum* and incubated at 22 °C under constant illumination of 60 μmol photons m^−2^ s^−1^. After 12 days, cells were washed off the plates and re-plated on an RSE-agar medium without norflurazon and incubated under constant illumination of 60 μmol photons m^−2^ s^−1^ for 12 days at 22 °C to test for recovery of viable cells. In addition, cells were stained with 10 µL of Aniline Blue (2.5% in 2% acetic acid; Millipore-Sigma, St. Louis, USA) and observed under a fluorescent microscope to ascertain damage to the cells^63^.

### *In vivo* UV-mutagenesis of WT *P. tricornutum* and selection of norflurazon-resistant mutants

The UV-mutagenesis of *P*. *tricornutum* cells was performed on a RSE-agar medium. Cells (3 × 10^7^ cells) from an exponentially growing culture were spread uniformly onto a 90-mm diameter Petri dish and allowed to dry. The lid was removed, and cells were mutagenized by direct irradiation of the agar surface with UVB rays from a UV-transilluminator (312 nm; Wilber Lourmant, France) for 15 s, 30 s, 1 min, 1.5 min and 2 min. UV-irradiated plates were left for 24 h recovery at an illumination of 30 μmol photons m^−2^ s^−1^ at 22 °C before re-plating the cells on a RSE-agar plate supplemented with 15 μM norflurazon. After 15 days, obtained colonies were re-streaked onto a fresh RSE-agar plate supplemented with 15 μM norflurazon, and the two fastest growing norflurazon-resistant lines were selected for further characterization.

### Preliminary determination of herbicide tolerance of norflurazon-resistant mutants

Mutants, cultured in liquid RSE medium until the mid-log phase, were diluted to 5 × 10^5^ cells mL^−1^ and transferred to a 24-well plate and challenged with norflurazon in the range of 10–40 μM. These cultures were incubated in an incubator shaker (120 rpm) at 18 °C and illumination of 75 μmol photons m^−2^ s^−1^, in an atmosphere enriched with CO_2_ (200 mL min^−1^). After 20 days incubation, tolerance to norflurazon was determined by visual observation. Preliminary experiments under the similar setup showed that these norflurazon concentrations are lethal for WT *P*. *tricornutum*.

### Comparative growth characterization of WT and mutants with and without norflurazon

WT and mutant (M1 and M2) cultures, grown to mid-log phase, were diluted to a chlorophyll concentration of 2 mg L^−1^ and cultivated in cylindrical glass columns in a Multi-Cultivator MC 1000-OD (Photon Systems Instruments, Czech Republic) with different norflurazon concentrations (0, 5 and 10 μM) at 22 °C, a light intensity of 50 μmol photons m^−2^ s^−1^, and bubbling with filtered air enriched with 2% CO_2_. Chlorophyll and cell density were recorded *in situ*, every 10 minutes for 93 h, via optical density measurements, at 680 nm and 720 nm, respectively. The experiment was repeated three times, and the average values are reported. At the end of the experiment, cells were observed microscopically under a bright field and were also stained with Aniline Blue to assess cellular damage.

### Phylogeny of the *P. tricornutum* phytoene desaturase-1 enzyme

Homologs of the *P*. *tricornutum* phytoene desaturase (PtPDS1; EEC48362.1) were searched against the NCBI database using the BLASTp algorithm^64^ and chloroplastic phytoene desaturase/dehydrogenase peptide sequences, representing a diverse phylogenetic group. Alignments were made using EMBL-EBI Clustal Omega^65^. Phylogenetic trees were built and tested using the ATGC: PhyML 3.0 server^66^ with Smart Model Selection^67^. Within PhyML 3.0, BIONJ^68^ was used to build a bootstrap tree with Subtree-Pruning-Regrafting^69^; tree improvements and branch support bootstrapping were set to 1000. Species with bootstrap values below 500 were iteratively dropped to arrive at the final phylogenetic tree.

### Cloning of the PtPDS1 gene

*P*. *tricornutum* genomic DNA was isolated using the Quick-DNA Plant/Seed Kit (Zymo Research, Irvine, USA) following the manufacturer’s instructions. The PtPDS1 CDS (PHATRDRAFT_45735; JGI database *P*. *tricornutum* v2.0) was PCR amplified from the genomic DNA of two mutants, designated M1 and M2, and WT as a reference, by Phusion HotStart II DNA polymerase (ThermoFisher Scientific, USA) with primers PtPDS1-F (5′-ATGATGTTTCACTATAAGACAGGGTCGTCATG-3′) and PtPDS1-R (5′-CTAGGCTTCCACGAATTGACTAGGATCAAC-3′). The PCR product was gel-eluted (Zymoclean™ Gel DNA Recovery Kit, Irvine, USA), ligated into a pJET1.2 vector (ThermoFisher Scientific, USA) and transformed into *E*. *coli*. Plasmids from six individual colonies were Sanger sequenced using pJET1.2-F (5′-CGACTCACTATAGGGAGAGCGGC-3′), pJET1.2-R (5′-AAGAACATCGATTTTCCATGGCAG-3′) and PtPDS1-Seq-F (5′ TATTCTCATGAACCAGAAAATGCTCACTTTGC-3′) primers. Sequencing chromatograms were aligned to the PHATRDRAFT_45735 coding sequence in Benchling (https://benchling.com) to identify mutations.

### Secondary and tertiary structure analysis of PtPDS1 peptide sequence

The PtPDS1 tertiary structure was modeled on *Oryza sativa* phytoene desaturase-1 (OsPDS1; PDB: 5MOG.A) with SWISS-MODEL^70^. Secondary structure alignments rendered above the amino acid sequence alignment, using the ESPript 3.0 server^71^. The modeled tertiary structure of PtPDS1 was superimposed on the OsPDS1 chain A complexed with norflurazon, and the effect of amino acid substitution leading to norflurazon tolerance was analyzed using Coot^72^.

### Vector construction of pPtPDS1-M1 and pBS-PtPDS1-M1

A pPtPDS1-M1 (GenBank accession MK645852) vector was generated by replacing s*h-ble* CDS in pPha-T1::eGFP (a kind gift from Prof. Assaf Vardi) with PtPDS1-M1 CDS. The pPha-T1::eGFP vector digested with *XhoI* (3967 bp) served as a vector backbone to assemble fcpB-Prom (fcpB-Prom-F 5′-cacaggatgagttttctcgaACTAGTACATACCTTCAGCGTCGTCTTCACTG-3′ + fcpB-Prom-R 5′-gtcttatagtgaaacatcatCTTGACATCTGGCAACCGTGA-3′), PtPDS1-M1 (inf-PtPDS1-F 5′-cacggttgccagatgtcaagATGATGTTTCACTATAAGACAGGG-3′ + inf-PtPDS1-R attaaatttttaaggaaggtCTAGGCTTCCACGAATTGAC) and fcpA-Term (fcpA-Term-F 5′-gtcaattcgtggaagcctagACCTTCCTTAAAAATTTAATTTTCATTAGTTGCA-3′ + fcpA-Term-R 5′-tatagggagaccggcctcgaACTAGTCTCGAGAAAACTCATCCTGTGCCTTC-3′) fragments in a one-pot InFusion cloning reaction (Takara-Clontech, USA). Underlined sequences within a primer pair were used for annealing and amplification of the desired template with overlapping complementary overhangs (indicated in lower case). *SpeI* restriction sites (5′-ACTAGT-3′) were incorporated in primers flanking the PtPDS1-M1 expression cassette for easy sub-cloning into the vector of choice.

The pBS-PtPDS1-M1 (GenBank Accession MK645853) vector was generated by *SpeI* excision of the PtPDS1-M1 expression cassette from the pPtPDS1-M1 vector and ligation into the respective site in pBlueScript II SK (+) plasmid (Agilent, Cat#212205). The cloning strategy was conceptualized in Benchling (https://benchling.com), and vector maps were generated in SnapGene Viewer 4.0.4 (http://www.snapgene.com).

### Biolistic transformation of *P. tricornutum*

The WT *P*. *tricornutum* strain UTEX 646 culture in mid-log phase was diluted with an equal volume of RSE medium and grown for another three days. On the day of transformation, cells were pelleted by centrifugation (2200 × g for 5 min), and 3 × 10^7^ cells were spread on each RSE-agar plate. The plates were left to dry in the laminar hood for 1.5 h before particle bombardment. Tungsten-M17 particles (Bio-Rad) were coated with 5 µg of DNA, as per the manufacturer’s instructions. Each DNA-coated micro-projectile preparation was spent in two shots. Biolistic gene delivery was performed using the PDS-1000/He™ gene gun (Bio-Rad, Hercules, USA); shots were fired at 1350 psi under a vacuum of 27 in. Hg at a shelf distance of 6 cm^22^. Bombarded cells were incubated at 22 °C under constant illumination of 30 μmol photons m^−2^ s^−1^ for 24 h to recover. Post-recovery, cells were re-plated on ½ × RSE-agar + 3 or 5 μM norflurazon and illuminated at 60 μmol photons m^−2^ s^−1^ at 22 °C for 12 days to allow the selection of transgenic events.

### Calculation of transformation efficiency with pPtPDS1-M1 and statistical analysis of transformation optimization

All putative transgenic events from five plasmid-microcarrier coating reactions each, shot and selected on 3 or 5 µM norflurazon plates, were screened by PCR amplification for eGFP and PtPDS1-M1 integration.

Transformation efficiency was calculated as follows:

$${\rm{Transformation}}\,{\rm{efficiency}}\,( \% )=\tfrac{{\rm{Total}}\,{\rm{number}}\,{\rm{of}}\,{\rm{PtPDS}}1-{\rm{M}}1/{\rm{eGFP}}\,{\rm{PCR}}\,{\rm{positive}}\,\mathrm{events}\,}{{\rm{Total}}\,{\rm{number}}\,{\rm{of}}\,{\rm{events}}\,{\rm{screened}}}\times 100$$

$${\rm{F}}{\rm{a}}{\rm{l}}{\rm{s}}{\rm{e}}\,{\rm{p}}{\rm{o}}{\rm{s}}{\rm{i}}{\rm{t}}{\rm{i}}{\rm{v}}{\rm{e}}\,({\rm{ \% }})=\frac{{\rm{T}}{\rm{o}}{\rm{t}}{\rm{a}}{\rm{l}}\,{\rm{n}}{\rm{u}}{\rm{m}}{\rm{b}}{\rm{e}}{\rm{r}}\,{\rm{o}}{\rm{f}}\,{\rm{P}}{\rm{t}}{\rm{P}}{\rm{D}}{\rm{S}}1-{\rm{M}}1\,{\rm{P}}{\rm{C}}{\rm{R}}\,{\rm{n}}{\rm{e}}{\rm{g}}{\rm{a}}{\rm{t}}{\rm{i}}{\rm{v}}{\rm{e}}\,{\rm{e}}{\rm{v}}{\rm{e}}{\rm{n}}{\rm{t}}{\rm{s}}\,}{{\rm{T}}{\rm{o}}{\rm{t}}{\rm{a}}{\rm{l}}\,{\rm{n}}{\rm{u}}{\rm{m}}{\rm{b}}{\rm{e}}{\rm{r}}\,{\rm{o}}{\rm{f}}\,{\rm{e}}{\rm{v}}{\rm{e}}{\rm{n}}{\rm{t}}{\rm{s}}\,{\rm{s}}{\rm{c}}{\rm{r}}{\rm{e}}{\rm{e}}{\rm{n}}{\rm{e}}{\rm{d}}}\times 100$$

Transformation optimization experiments were compared by t-test at α = 0.05 in Microsoft excel using Data AnalysisToolPak.

### Molecular characterization and validation of transgene expression

On-colony PCR screening of putative transgenic *P*. *tricornutum* cells was performed as described by Nymark *et al*.^26^. Insertion of PtPDS-M1 in *P*. *tricornutum* genomic DNA was confirmed using the primer pair seq-fcpB-P-F (5′-ACATACCTTCAGCGTCGTCTTCACTG-3′) + PtPDS1-R (5′-CTAGGCTTCCACGAATTGACTAGGATCAAC-3′); similarly, eGFP was confirmed using eGFP-F (5′-CGTAAACGGCCACAAGTTCAG-3′) + eGFP-R (5′-AACTCCAGCAGGACCATGTG-3′).

Southern hybridization of purified genomic DNA from transgenic lines was performed to confirm integration of pPtPDS1-M1 DNA in the *P*. *tricornutum* genome. A wet pellet weighing 3 g was lyophilized and subsequently used for high quality genomic DNA purification following the Dellaporta protocol^73^. *KpnI-*digested genomic DNA (9 µg) was resolved on a 1% agarose gel and neutral blotted to a HyBond-N + nylon membrane (GE Healthcare, Little Chalfont, UK) for Southern blotting using a DIG High Prime DNA Labelling and Detection Starter Kit I (Roche, Penzberg, Germany). Templates for DIG labeling were PCR amplified and gel-eluted. A template for the eGFP probe (606 bp) was amplified with eGFP-F and eGFP-R primers. Similarly, a template for the PtPDS1 probe (581 bp) was amplified with PtPDS1-F and PtPDS1-R primers, followed by the addition of *XhoI* restriction enzyme (New England BioLabs, Ipswich, USA) to the PCR reaction (incubated at 37 °C for 2 h), and gel-elution of a 581 bp fragment. DIG-labeled probes were hybridized at 54 °C overnight and stringency washed at 68 °C.

### Western blot analysis

Expression of eGFP in transgenic *P*. *tricornutum* lines was validated by probing a western blot of 20 µg crude protein extract resolved on a 10% SDS-PAGE gel with an anti-TAG(CGY)FP antibody (Evrogen, Moscow, Russia) at a dilution of 1:5000 and a secondary antibody, goat anti-rabbit IgG conjugated to horseradish peroxidase (Bio-Rad, Hercules, USA) at a dilution of 1:3000. Chemiluminescence development of the western blot bound antibody was done using an EZ-ECL Kit (Biological Industries, Beit Haemek, Israel) according to the manufacturer’s instructions and imaged with a MicroChemi detection system (DNR Bio-Imaging Systems Ltd., Jerusalem, Israel).

### Fluorescence microscopy

*In vivo* characterization of lines expressing eGFP was verified by fluorescence microscopy with filter sets 38 HE and 16 for visualization of eGFP and chlorophyll auto-fluorescence, respectively, using a Zeiss Imager A2 microscope (Carl Zeiss MicroImaging Inc., Germany) with a Zeiss AxioCamMRc mounted digital camera.

## Supplementary information

Supplementary data and figures
