# Supplementary material for: A novel endogenous selection marker for the diatom Phaeodactylum tricornutum based on a unique mutation in phytoene desaturase 1
Source: Sci Rep. 2019 Jun 3;9:8217. doi: 10.1038/s41598-019-44710-5 (PMC6546710; doi:10.1038/s41598-019-44710-5)
Supplement: Supplementary file 1 — Supplementary data and figures [file 41598_2019_44710_MOESM1_ESM.pdf]

# Supplementary Information File

## A novel endogenous selection marker for the diatom *Phaeodactylum tricornutum* based on a unique mutation in phytoene desaturase 1

Yogesh Taparia[1], Aliza Zarka[1], Stefan Leu[1], Raz Zarivach[2], Sammy Boussiba[1] and Inna Khozin-Goldberg[1*]

[1] Microalgal Biotechnology Laboratory, The Jacob Blaustein Institutes for Desert Research, Ben-Gurion University of the Negev, Midreshet Ben-Gurion, Israel, 849900

[2] Department of Life Sciences, Faculty of Natural Sciences, Ben-Gurion University of the Negev, Beer-Sheva, Israel, 8410501

***Corresponding Author:**
Inna Khozin-Goldberg
khozin@bgu.ac.il

Figure S1: Aniline Blue staining of norflurazon treated WT *P. tricornutum* cells.

Figure S2: Phylogenetic analysis of Phytoene desaturase peptide sequences across various phylogenetic species.

Figure S3. Sequence analysis of phytoene desaturase-1 from *P. tricornutum*.

Figure S4. Vector maps

Figure S5. PCR Screening of transgenic lines.

Figure S6. Evaluation of long-term stability of transgenic lines.

Full length Southern blot from figure 5b

Full length Western blot from figure 5c

*Phaeodactylum tricornutum* UTEX646 PDS1 CDS of WT, M1 and M2 obtained by Sanger sequencing (FASTA formatted)

pPtPDS1-M1 vector Sequence (GenBank accession MK645852)

pBS-PtPDS1-M1 vector Sequence (GenBank accession MK645853)

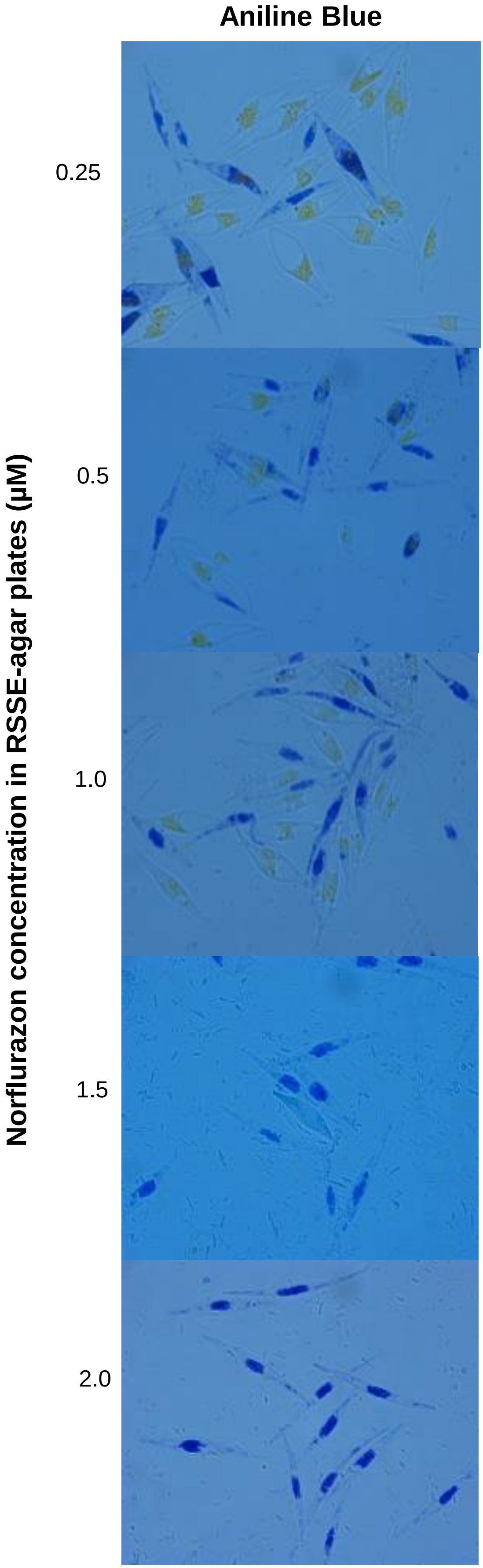


**Fig S1.** Aniline Blue staining of norflurazon treated WT *P. tricornutum* cells. $3\times10^{7}$ WT cells were plated on RSE-agar supplemented with Norflurazon concentrations ranging from 0.25-5µM. Plated cells were incubated in light and washed off after 12 days of incubation and stained with Aniline Blue for microscopic evaluation of cell viability.

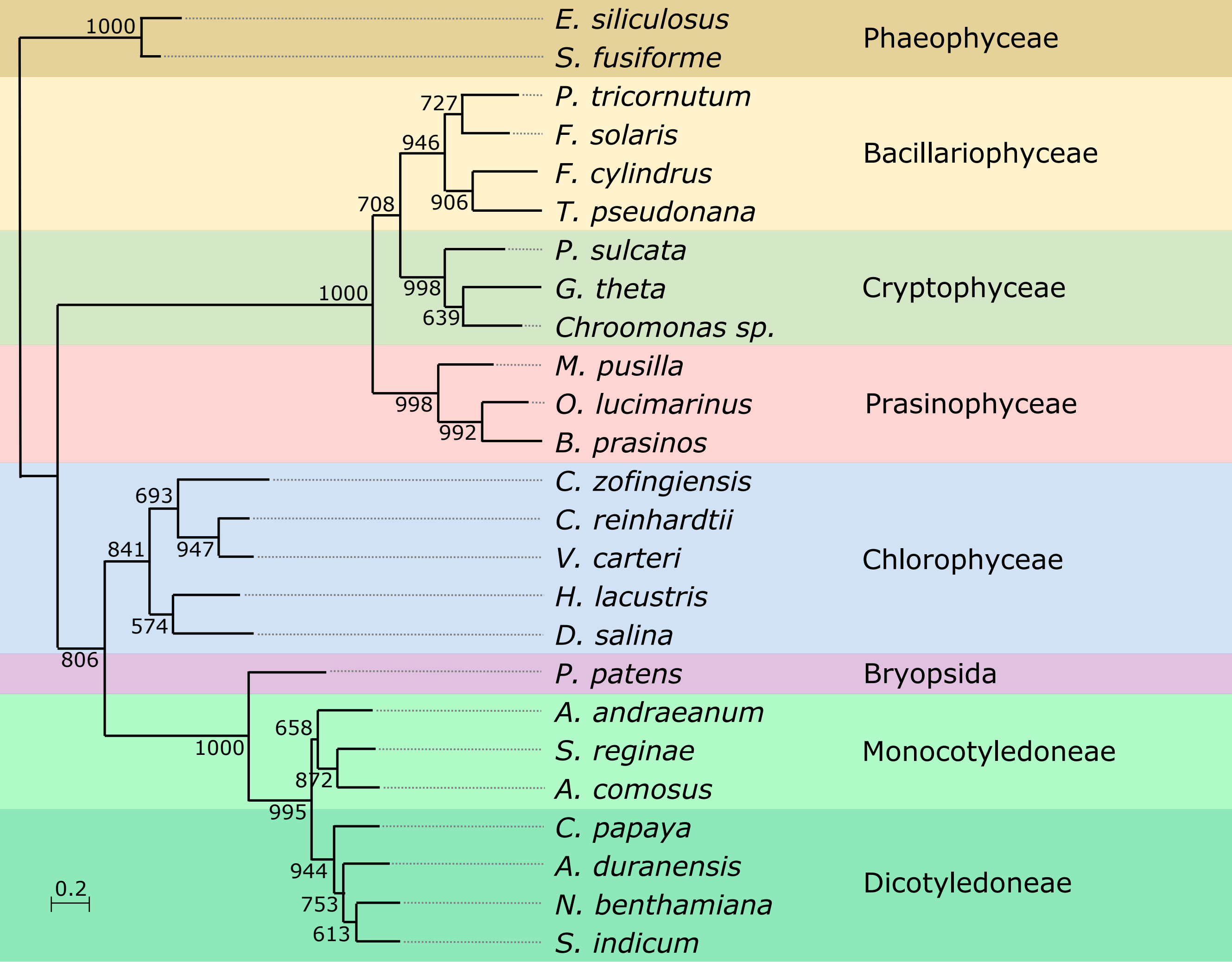


**Fig S2.** Phylogenetic analysis of Phytoene desaturase peptide sequences across various phylogenetic species. Peptide sequences were obtained from NCBI database and aligned using default settings in Clustal Omega. Phylogenetic tree construction and bootstrap analysis was performed using the PhyML 3.0 server and Akine Information Criterion enabled Smart Model Selection. Best substitution model was LG+G+I. Initial trees were constructed using the BIONJ algorithm with Subtree-Pruning-Regrafting based tree improvement. Branch length and bootstrap values were obtained from of 1000 replicates using maximum likelihood algorithm. Branch nodes with bootstrap values greater than 500 are labeled. Scale bar represents 0.2 substitutions per amino acid residue. *Ectocarpus siliculosus* (CBN77338.1), *Sargassum fusiforme* (AUE44545.1), *Phaeodactylum tricornutum* CCAP 1055/1 (EEC48362.1), *Fistulifera solaris* (GAX13863.1), *Fragilariopsis cylindrus* CCMP1102 (OEU19277.1), *Thalassiosira pseudonana* CCMP1335 (EED91739.1), *Proteomonas sulcata* (AUE44558.1), *Guillardia theta* CCMP2712 (EKX49047.1), *Chroomonas* sp. (AUE44559.1), *Micromonas pusilla* CCMP1545 (EEH60398.1), *Ostreococcus lucimarinus* CCE9901 (ABO98307.1), *Bathycoccus prasinos* (CCO18703.1), *Chromochloris zofingiensis* (ABR20878.1), *Chlamydomonas reinhardtii* (EDP05305.1), *Volvox carteri* f. *nagariensis* (EFJ50562.1), *Haematococcus lacustris* (AAV37090.1), *Dunaliella salina* (ADD52599.1), *Physcomitrella patens* (EDQ51647.1), *Anthurium andraeanum* (AML60257.1), *Strelitzia reginae*, (AGC74040.1), *Ananas comosus* (OAY66714.1), *Carica papaya*, (ABG72807.2), *Arachis duranensis* (XP_015958360.1), *Nicotiana benthamiana* (ABY25272.1) and *Sesamum indicum* (AHV90407.1).

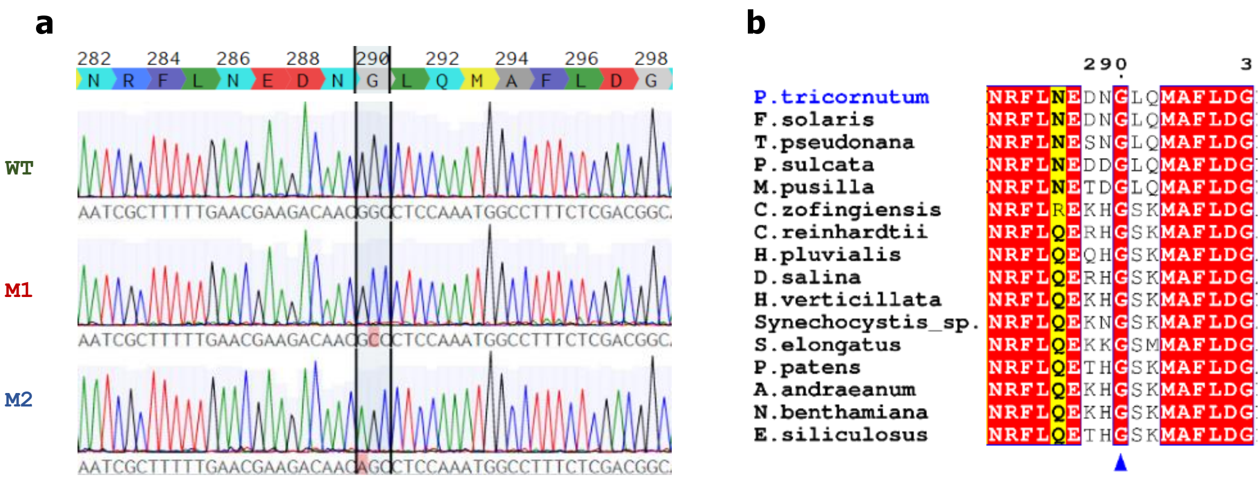


**Fig. S3.** Sequence analysis of phytoene desaturase-1 from *P. tricornutum*. (**a)** Alignment of sanger sequencing reads from WT, M1 and M2 lines. Resistance to the bleaching herbicide norflurazon is encoded in single point mutations occurring in codon 290 leading to amino acid change from Glycine (WT) to Ala (M1) or Ser (M2). (**b)** Partial alignment of phytoene desaturase peptide sequences from diverse phylogenetic groups confirms Gly290 (blue triangle) is a highly conserved residue.

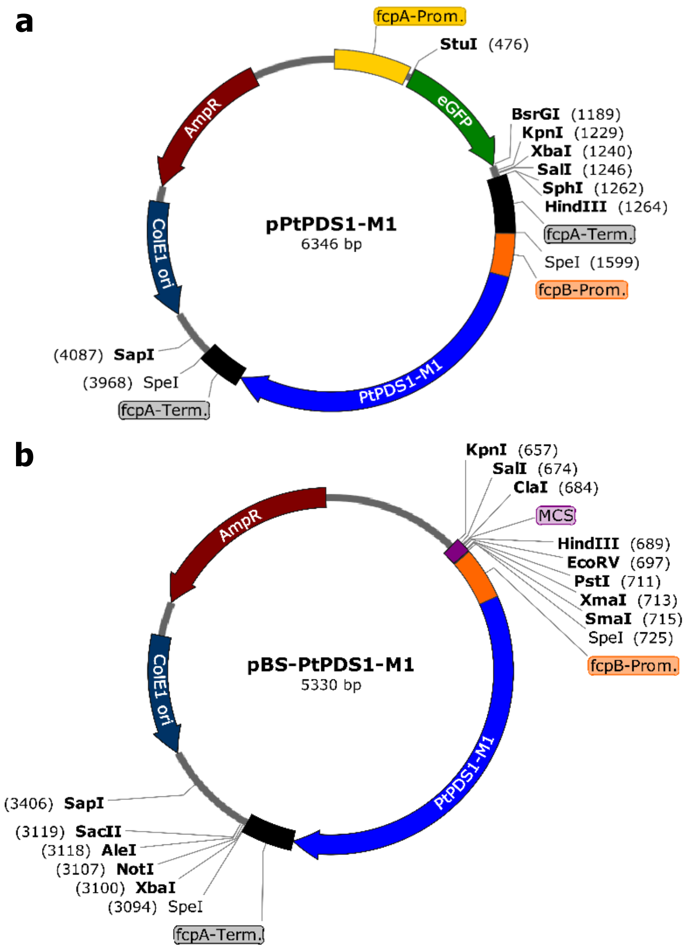


**Fig. S4.** Vector maps of (**a**) pPtPDS1-M1 (GenBank accession MK645852) vector contains eGFP driven by the *fcpA* promoter and the mutant PtPDS1-M1 driven by *fcpB* promoter constituting reporter and selectable marker cassettes respectively, was used to transform and validate a new transformation system in *P. tricornutum* UTEX 646. (**b**) pBS-PtPDS1-M1 (GenBank accession MK645853 was constructed by excision of the PtPDS1-M1 expression cassette flanked by *SpeI* restriction sites from pPtPDS1-M1 vector and ligation into the *SpeI* site of  pBlueScript-SK II (+) plasmid.

M1 and M2 = Mutants
WT = Wild-type
T1-4 = Stable transgenic events
US = Unstable line

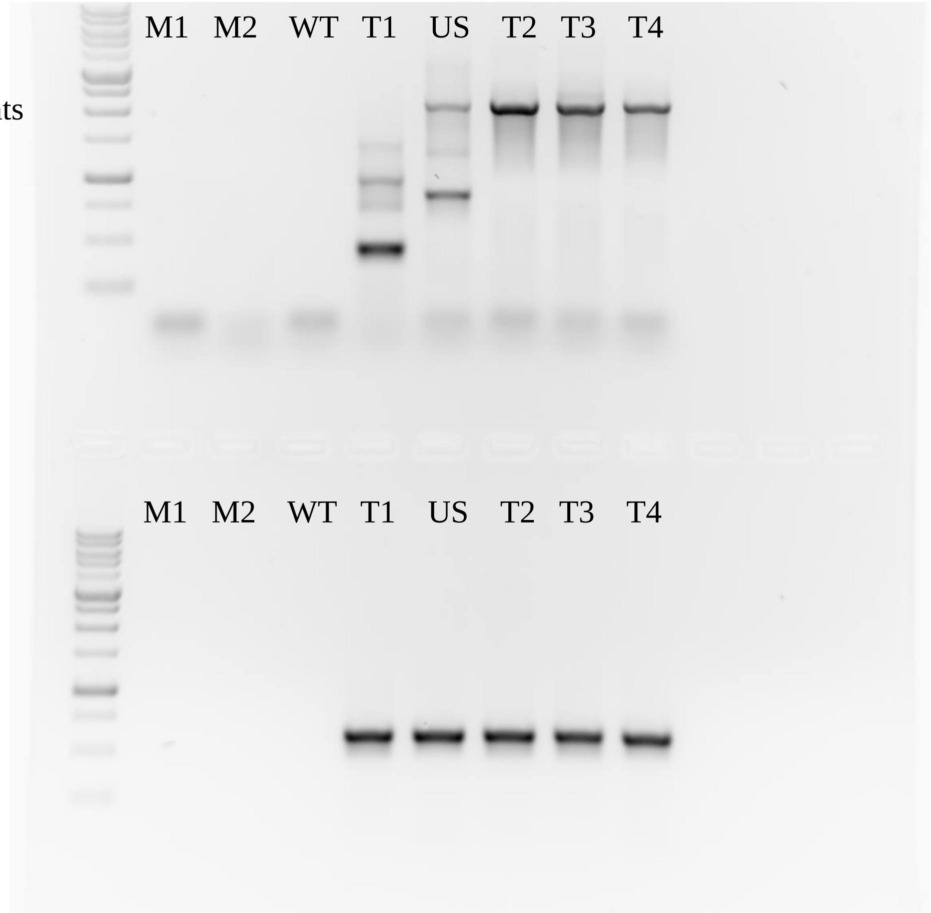


Fig S5. PCR Screening of transgenic lines.

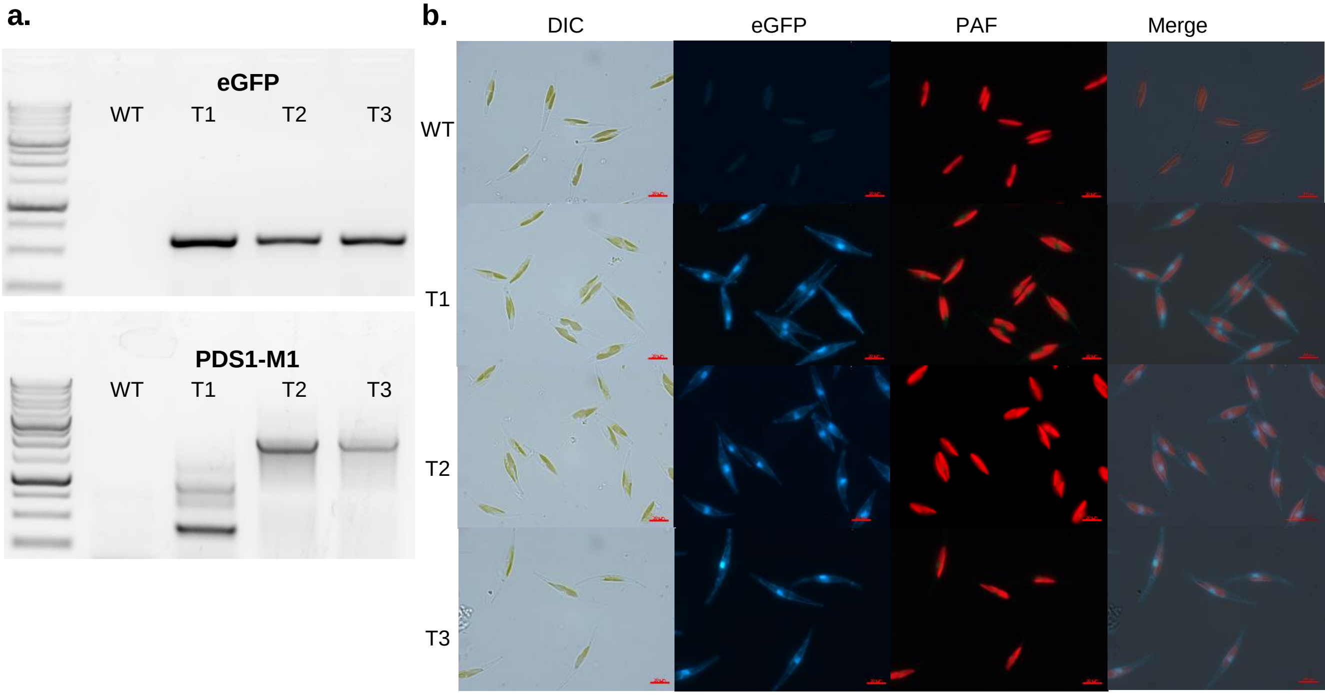


**Fig S6.** Stability evaluation of maintained transgenic lines after 48 months. **a.** Stability of transgenic lines with different insert copy number were selected for PCR screened of eGFP and PDS1-M1. **b.** Verification of eGFP expression by fluorescent microscopy. DIC-differential interference contrast; eGFP-enhanced green fluorescent protein; PAF-plastidial auto-fluorescence.

# Full Length Southern Blot (Figure 5b)

M1 and M2 = Mutants
WT = Wild-type
T1-4 = Stable transgenic events
US = Unstable line

PDS
1

M1 M2 WT T1 US T2 T3 T4

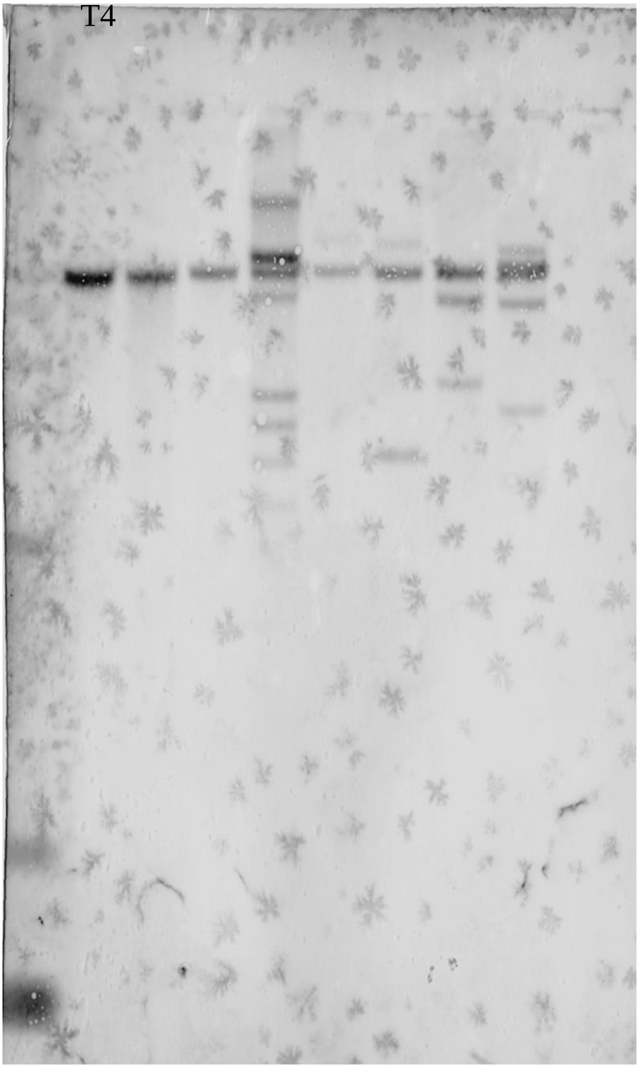

eGF
P

M1 M2 WT T1 US T2 T3 T4

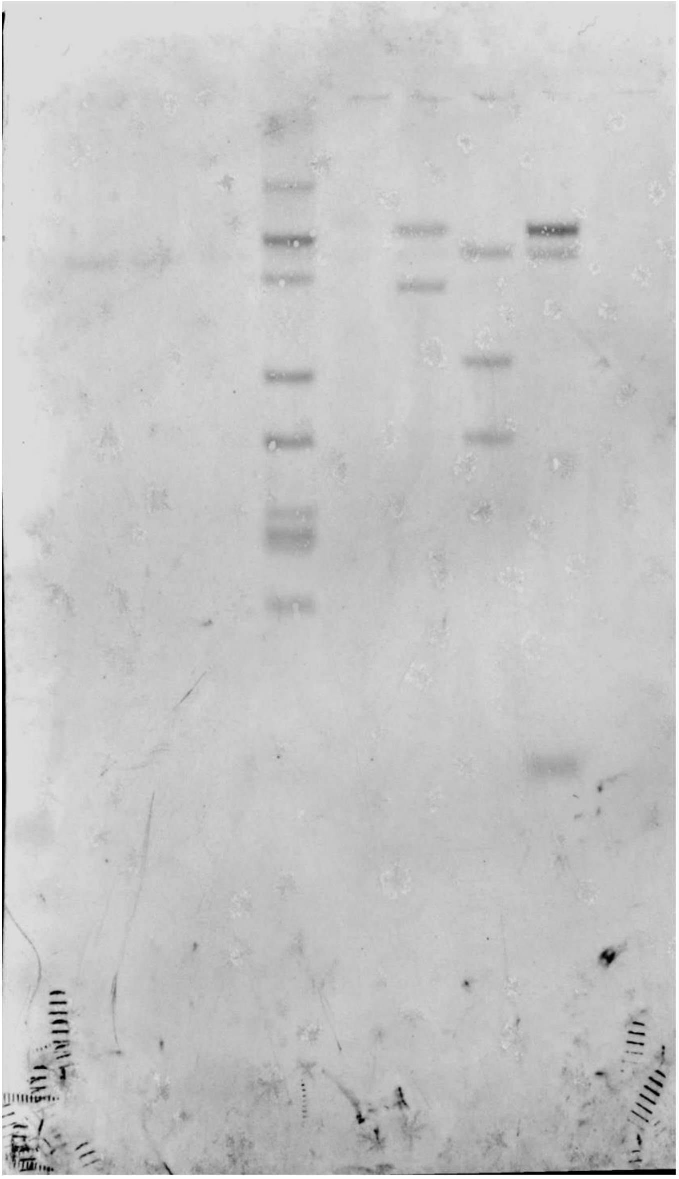

# Full Length Western Blot (Fig 5c)

M1 and M2 = Mutants
WT = Wild-type
T1-4 = Stable transgenic events
US = Unstable line

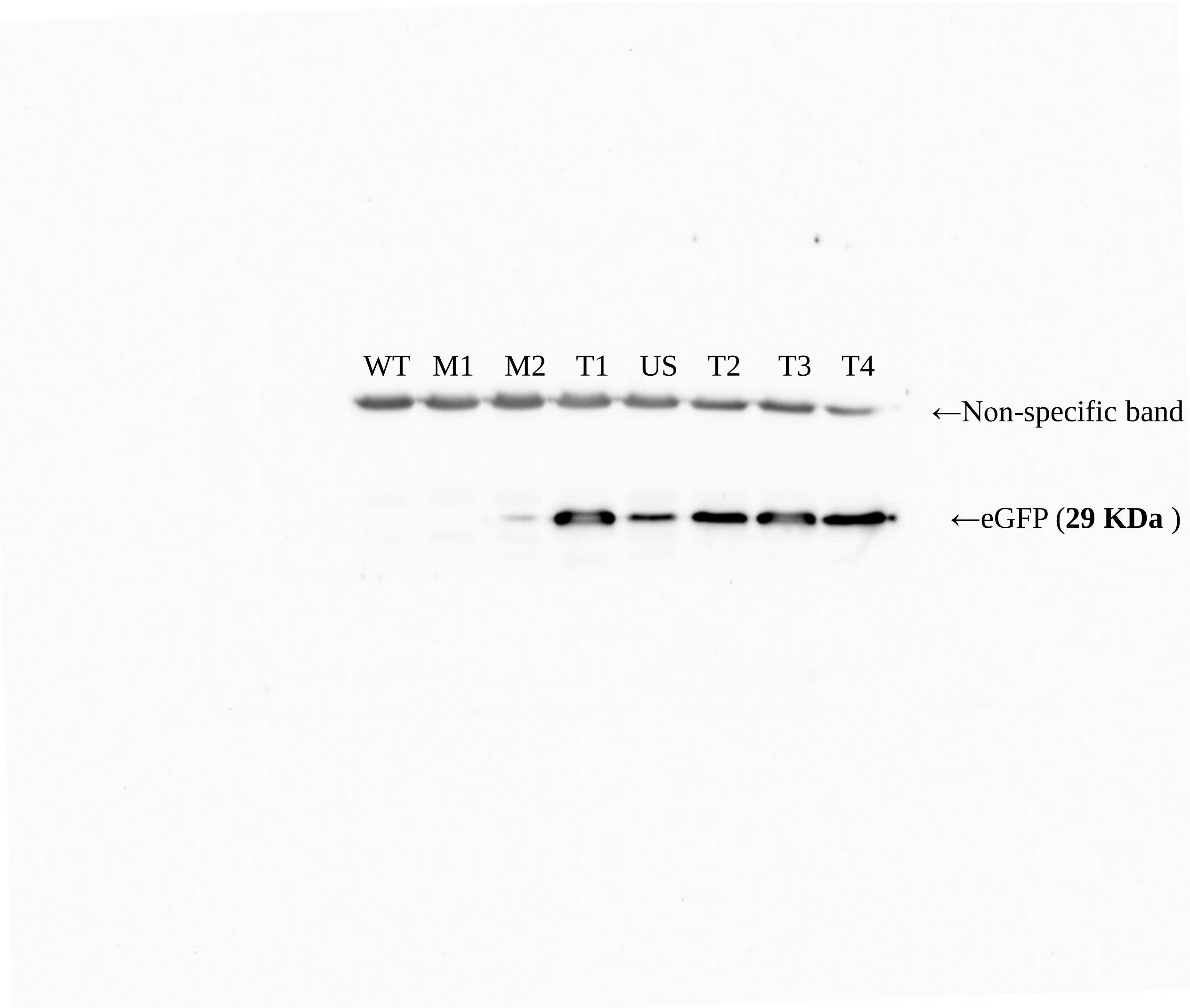

PtPDS1 CDS of WT, M1 and M2 obtained by Sanger sequencing

Mutated codon (amino acid residue 290) is highlighted in the respective sequence.

>PtPDS1-WT_UTEX646

ATGATGTTTCACTATAAGACAGGGTCGTCATGGTTCCTGCTGCTGTCCGCATCCATTACCACAACCCTCACGACGACAA
CGATcACAACAACCCACGCCTTTGCCCCGCACACGCGCTTATCCGTGCCCCACGGTGCTTCCCGACTCGTCATGAAGGA
TTTCCCCAAACCCAATTTGGAAGATACGGACAATTACCGTTTGTATCGTGATTTGTCGCACTCCTTCTCCACGACACTC
AAGGCACCGTCGCCAGAATCACGCAAGAAGGTAGCCATCATTGGCGGTGGCTTGTCCGGTCTCGCCTGTGCCAAGTAC
CTCGCCGACGCCGGACACCAACCCGTCGTCTACGAAGCACGGGACGTCCTCGGCGGCAAGGTCTCCGCTTGGCAAGAT
GCCGACGGAGACTGGATCGAAACCGGACTACACATTTTCTTTGGTGCCTACCCGAATATGATGAATCTCTTTGCCGAAC
TTGATATTCACGATCGTCTCCAGTGGAAGGTGCACAAAATGATCTTTGCCATGCAGGAACTACCCGGAGAATTCACAA
CCTTTGATTTCATTCCCGGCATACCCGCACCCTTCAATTTCGGACTCGCTATTCTCATGAACCAGAAAATGCTCACTTTG
CCGGAGAAAATACAGACGGCTCCCCCGCTACTGCCCATGCTCGTACGCGGACAAGACTTTATCGACGAACAGGACGAA
CTCTCCGTCCTCGACTTTATGCGCAAGTACGGGATGCCCGAACGCATCAACGAAGAAGTCTTTATCAGCATGGCCAAG
GCACTCGATTTCATTGATCCCGACAAATTGAGTATGACGGTCGTTTTGACCGCCATGAATCGCTTTTTGAACGAAGACA
ACGGCCTCCAAATGGCCTTTCTCGACGGCAACCAGCCCGACCGGCTCTGTGCACCCATGGTCGAACACATTCAAGCCC
GTGGCGGTCAAGTCAACCTCAATTCCCCCGTGCAAGAAATCGTCACCCGCGAAGACGGTAGTGTCGATTACCTCCTGA
TGCGGTCCGGAGAAAAGGTCGTTGCCGACGAATACGTGTCCGCCATGCCCGTGGATATTGTCAAACGCATGTTGCCCG
AAAAGTGGCAAACAATGCCCTACTTTCGTCAATTCGACGAACTCGAAGGCATTCCCGTCATTAACCTCCACATGTGGTT
CGATCGCAAACTCAAAGCCGTCGATCACTTGTGCTTTTCCCGCTCGCCGCTGCTGTCCGTTTACGCCGACATGAGCGTC
ACCTGCAAGGAATACTACGACGAATCCGCTTCCATGCTCGAGCTCGTCTTTGCCCCGTGCAGTCCACTCGCCGGTGGCA
ACGTGAATTGGATTGCCAAGACGGACGAAGAAATCATCGACGCGACCATGGGCGAACTGGCCCGACTCTTTCCCACCG
AAATCGCCGCTGATCCCACCTGGCCGGCCACCAAGAACCAGGGCCCCAACGGTGAGGCCAAACTTCGCAAATACGCC
GTGGTCAAGGTGCCCCGCTCGGTCTACGCCGCCATTCCCGGCCGCAACAAGTACCGCCCGAGTCAAACCACTCCAATT
GACAATTTCACGCTGGCCGGGGACTGGACGTCACAAAAGTTTCTGGGATCCATGGAAGGCGCCGTCCTCGGGGGCAAG
CTCGCCGCCGAAGTATTGGCCCGCAAGGCGGCCAACCTACCCGCACCCGAACTCGCCAACAAACCCGTGCGGGATGAA
ATTGTGCAAAAGGCGCAAACACACGAAGCGCGTCCACCGGCCGGAGTCAAGGGACAAGGTGCGATTGCCTTTGGCGG
TGGCGCCGTGCTCGGGACGGAAAACAAAGCCTTGCTTCGGGACGTTGATCCTAGTCAATTCGTGGAAGCCTAG

>PtPDS1-M1_Gly290Ala

ATGATGTTTCACTATAAGACAGGGTCGTCATGGTTCCTGCTGCTGTCCGCATCCATTACCACAACCCTCACGACGACAA
CGATcACAACAACCCACGCCTTTGCCCCGCACACGCGCTTATCCGTGCCCCACGGTGCTTCCCGACTCGTCATGAAGGA
TTTCCCCAAACCCAATTTGGAAGATACGGACAATTACCGTTTGTATCGTGATTTGTCGCACTCCTTCTCCACGACACTC
AAGGCACCGTCGCCAGAATCACGCAAGAAGGTAGCCATCATTGGCGGTGGCTTGTCCGGTCTCGCCTGTGCCAAGTAC
CTCGCCGACGCCGGACACCAACCCGTCGTCTACGAAGCACGGGACGTCCTCGGCGGCAAGGTCTCCGCTTGGCAAGAT
GCCGACGGAGACTGGATCGAAACCGGACTACACATTTTCTTTGGTGCCTACCCGAATATGATGAATCTCTTTGCCGAAC
TTGATATTCACGATCGTCTCCAGTGGAAGGTGCACAAAATGATCTTTGCCATGCAGGAACTACCCGGAGAATTCACAA
CCTTTGATTTCATTCCCGGCATACCCGCACCCTTCAATTTCGGACTCGCTATTCTCATGAACCAGAAAATGCTCACTTTG
CCGGAGAAAATACAGACGGCTCCCCCGCTACTGCCCATGCTCGTACGCGGACAAGACTTTATCGACGAACAGGACGAA
CTCTCCGTCCTCGACTTTATGCGCAAGTACGGGATGCCCGAACGCATCAACGAAGAAGTCTTTATCAGCATGGCCAAG
GCACTCGATTTCATTGATCCCGACAAATTGAGTATGACGGTCGTTTTGACCGCCATGAATCGCTTTTTGAACGAAGACA
ACGCCCTCCAAATGGCCTTTCTCGACGGCAACCAGCCCGACCGGCTCTGTGCACCCATGGTCGAACACATTCAAGCCC
GTGGCGGTCAAGTCAACCTCAATTCCCCCGTGCAAGAAATCGTCACCCGCGAAGACGGTAGTGTCGATTACCTCCTGA
TGCGGTCCGGAGAAAAGGTCGTTGCCGACGAATACGTGTCCGCCATGCCCGTGGATATTGTCAAACGCATGTTGCCCG
AAAAGTGGCAAACAATGCCCTACTTTCGTCAATTCGACGAACTCGAAGGCATTCCCGTCATTAACCTCCACATGTGGTT
CGATCGCAAACTCAAAGCCGTCGATCACTTGTGCTTTTCCCGCTCGCCGCTGCTGTCCGTTTACGCCGACATGAGCGTC
ACCTGCAAGGAATACTACGACGAATCCGCTTCCATGCTCGAGCTCGTCTTTGCCCCGTGCAGTCCACTCGCCGGTGGCA
ACGTGAATTGGATTGCCAAGACGGACGAAGAAATCATCGACGCGACCATGGGCGAACTGGCCCGACTCTTTCCCACCG
AAATCGCCGCTGATCCCACCTGGCCGGCCACCAAGAACCAGGGCCCCAACGGTGAGGCCAAACTTCGCAAATACGCC
GTGGTCAAGGTGCCCCGCTCGGTCTACGCCGCCATTCCCGGCCGCAACAAGTACCGCCCGAGTCAAACCACTCCAATT
GACAATTTCACGCTGGCCGGGGACTGGACGTCACAAAAGTTTCTGGGATCCATGGAAGGCGCCGTCCTCGGGGGCAAG
CTCGCCGCCGAAGTATTGGCCCGCAAGGCGGCCAACCTACCCGCACCCGAACTCGCCAACAAACCCGTGCGGGATGAA
ATTGTGCAAAAGGCGCAAACACACGAAGCGCGTCCACCGGCCGGAGTCAAGGGACAAGGTGCGATTGCCTTTGGCGG
TGGCGCCGTGCTCGGGACGGAAAACAAAGCCTTGCTTCGGGACGTTGATCCTAGTCAATTCGTGGAAGCCTAG

>PtPDS1-M2_Gly290Ser

ATGATGTTTCACTATAAGACAGGGTCGTCATGGTTCCTGCTGCTGTCCGCATCCATTACCACAACCCTCACGACGACAA
CGATGACAACAACCCACGCCTTTGCCCCGCACACGCGCTTATCCGTGCCCCACGGTGCTTCCCGACTCGTCATGAAGGA
TTTCCCCAAACCCAATTTGGAAGATACGGACAATTACCGTTTGTATCGTGATTTGTCGCACTCCTTCTCCACGACACTC
AAGGCACCGTCGCCAGAATCACGCAAGAAGGTAGCCATCATTGGCGGTGGCTTGTCCGGTCTCGCCTGTGCCAAGTAC
CTCGCCGACGCCGGACACCAACCCGTCGTCTACGAAGCACGGGACGTCCTCGGCGGCAAGGTCTCCGCTTGGCAAGAT
GCCGACGGAGACTGGATCGAAACCGGACTACACATTTTCTTTGGTGCCTACCCGAACATGATGAATCTCTTTGCCGAAC
TTGATATTCACGATCGTCTCCAGTGGAAGGTGCACAAAATGATCTTTGCCATGCAGGAACTACCCGGAGAATTCACAA
CCTTTGATTTCATTCCCGGCATACCCGCACCCTTCAATTTCGGACTCGCTATTCTCATGAACCAGAAAATGCTCACTTTG
CCGGAGAAAATACAGACGGCTCCCCCGCTACTGCCCATGCTCGTACGCGGACAAGACTTTATCGACGAACAGGACGAA
CTCTCCGTCCTCGACTTTATGCGCAAGTACGGGATGCCCGAACGCATCAACGAAGAAGTCTTTATCAGCATGGCCAAG
GCACTCGATTTCATTGATCCCGACAAATTGAGTATGACGGTCGTTTTGACCGCCATGAATCGCTTTTTGAACGAAGACA
ACAGCCTCCAAATGGCCTTTCTCGACGGCAACCAGCCCGACCGGCTCTGTGCACCCATGGTCGAACACATTCAAGCCC
GTGGCGGTCAAGTCAACCTCAATTCCCCCGTGCAAGAAATCGTCACCCGCGAAGACGGTAGTGTCGATTACCTCCTGA
TGCGGTCCGGAGAAAAGGTCGTTGCCGACGAATACGTGTCCGCCATGCCCGTGGATATTGTCAAACGCATGTTGCCCG
AAAAGTGGCAAACAATGCCCTACTTTCGTCAATTCGACGAACTCGAAGGCATTCCCGTCATTAACCTCCACATGTGGTT
CGATCGCAAACTCAAAGCCGTCGATCACTTGTGCTTTTCCCGCTCGCCGCTGCTGTCCGTTTACGCCGACATGAGCGTC
ACCTGCAAGGAATACTACGACGAATCCGCTTCCATGCTCGAGCTCGTCTTTGCCCCGTGCAGTCCACTCGCCGGTGGCA
ACGTGAATTGGATTGCCAAGACGGACGAAGAAATCATCGACGCGACCATGGGCGAACTGGCCCGACTCTTTCCCACCG
AAATCGCCGCTGATCCCACCTGGCCGGCCACCAAGAACCAGGGCCCCAACGGTGAGGCCAAACTTCGCAAATACGCC
GTGGTCAAGGTGCCCCGCTCGGTCTACGCCGCCATTCCCGGCCGCAACAAGTACCGCCCGAGTCAAACCACTCCAATT
GACAATTTCACGCTGGCCGGGGACTGGACGTCACAAAAGTTTCTGGGATCCATGGAAGGCGCCGTCCTCGGGGGCAAG
CTCGCCGCCGAAGTATTGGCCCGCAAGGCGGCCAACCTACCCGCACCCGAACTCGCCAACAAACCCGTGCGGGATGAA
ATTGTGCAAAAGGCGCAAACACACGAAGCGCGTCCACCGGCCGGAGTCAAGGGACAAGGTGCGATTGCCTTTGGCGG
TGGCGCCGTGCTCGGGACGGAAAACAAAGCCTTGCTTCGGGACGTTGATCCTAGTCAATTCGTGGAAGCCTAG

```
LOCUS       Exported                6364 bp ds-DNA     circular SYN 30-OCT-2018
DEFINITION  pPtPDS1-M1 Phaeodactylum tricornutum nuclear transformation vector expressing
eGFP and conferring resistance to norflurazon
ACCESSION   MK645852
VERSION     .
KEYWORDS    .
SOURCE      synthetic DNA construct
  ORGANISM  synthetic DNA construct
REFERENCE   1  (bases 1 to 6364)
  AUTHORS   Yogesh Taparia, Aliza Zarka, Stefan Leu, Raz Zarivach, Sammy
            Boussiba, Inna Khozin-Goldberg
  TITLE     A novel endogenous selection marker for the diatom Phaeodactylum
            tricornutum based on a unique mutation in phytoene desaturase 1
            confers resistance to norflurazon
  JOURNAL   Scientific Reports
FEATURES             Location/Qualifiers
     source          1..6364
                     /organism="synthetic DNA construct"
                     /mol_type="other DNA"
     promoter        478..912
                     /label=fcpA-Prom
                     /label=fcpA-P
     CDS             939..1658
                     /codon_start=1
                     /label=eGFP
                     /label=GFP2
                     /translation="MVSKGEELFTGVVPILVELDGDVNGHKFSVSGEGEGDATYGKLTL
                     KFICTTGKLPVPWPTLVTTLTYGVQCFSRYPDHMKQHDFFKSAMPEGYVQERTIFFKDD
                     GNYKTRAEVKFEGDTLVNRIELKGIDFKEDGNILGHKLEYNYNSHNVYIMADKQKNGIK
                     VNFKIRHNIEDGSVQLADHYQQNTPIGDGPVLLPDNHYLSTQSALSKDPNEKRDHMVLL
                     EFVTAAGITLGMDELYK"
     terminator      1729..2057
                     /label=fcpA Term
                     /label=fcpA-Term
     promoter        2067..2312
                     /label=fcpB-Prom
     CDS             2321..4195
                     /codon_start=1
                     /label=PtPDS1-M1
                     /translation="MMFHYKTGSSWFLLLSASITTTLTTTTITTTHAFAPHTRLSVPHG
                     ASRLVMKDFPKPNLEDTDNYRLYRDLSHSFSTTLKAPSPESRKKVAIIGGGLSGLACAK
                     YLADAGHQPVVYEARDVLGGKVSAWQDADGDWIETGLHIFFGAYPNMMNLFAELDIHDR
                     LQWKVHKMIFAMQELPGEFTTFDFIPGIPAPFNFGLAILMNQKMLTLPEKIQTAPPLLP
                     MLVRGQDFIDEQDELSVLDFMRKYGMPERINEEVFISMAKALDFIDPDKLSMTVVLTAM
                     NRFLNEDNALQMAFLDGNQPDRLCAPMVEHIQARGGQVNLNSPVQEIVTREDGSVDYLL
                     MRSGEKVVADEYVSAMPVDIVKRMLPEKWQTMPYFRQFDELEGIPVINLHMWFDRKLKA
                     VDHLCFSRSPLLSVYADMSVTCKEYYDESASMLELVFAPCSPLAGGNVNWIAKTDEEII
                     DATMGELARLFPTEIAADPTWPATKNQGPNGEAKLRKYAVVKVPRSVYAAIPGRNKYRP
                     SQTTPIDNFTLAGDWTSQKFLGSMEGAVLGGKLAAEVLARKAANLPAPELANKPVRDEI
                     VQKAQTHEARPPAGVKGQGAIAFGGGAVLGTENKALLRDVDPSQFVEA"
     terminator      4202..4443
                     /label=fcpA-Term
     rep_origin      complement(4741..5329)
                     /direction=LEFT
                     /label=ori
                     /note="high-copy-number ColE1/pMB1/pBR322/pUC origin of
                     replication"
     CDS             complement(5500..6364)
                     /label=AmpR
                     /label=Amp
ORIGIN
        1 ttccttttc aatattattg aagcatttat cagggttatt gtctcatgag cggatacata
       61 tttgaatgta tttagaaaaa taaacaaata ggggttccgc gcacatttcc ccgaaaagtg
      121 ccacctgacg tctaagaaac cattattatc atgacattaa cctataaaaa taggcgtatc
      181 acgaggccct ttcgtctcgc gcgtttcggt gatgacggtg aaaacctctg acacatgcag
      241 ctcccggaga cggtcacagc ttgtctgtaa gcggatgccg ggagcagaca agcccgtcag
      301 ggcgcgtcag cgggtgttgg cgggtgtcgg ggctggctta actatgcggc atcagagcag
      361 attgtactga gagtgcacca tatggacata ttgtcgttag aacgcggcta caattaatac
      421 ataaccttat gtatcataca catacgattt aggtgacact atagaaccag atcccccggg
      481 ctgcaggacg caatggagga ttatcaccgc aaaaatgaac ttcgaaaaaa actttcgagc
      541 gaccatggaa aaggaggatc agattcagat tacaacagtg gattgctctg gtagcaaata
      601 tcttctgcta gattggctca tggtcggttt tggacgttcg aagctcaccg tcaaaagaaa
      661 caaaagagaa gaatgacgtc ttcgtgacgt agaatctacg actgtactcg gatctgggaa
      721 atgaattgac tcacggtctt cttcgagtcc tgttacaggc ccttggtccg aacccccaca
      781 cgatttttgc accaaagatt tgcttcaatt tgctggatgt tttgactgca agatcagctg
      841 gcctagcaag agtgctcgtg ttgcttcgtc gggaatccct acgaatttca gttctgcaca
      901 aatttgtctg ccgtttcgag aattcgatcg tcaggcctat ggtgagcaag ggcgaggagc
      961 tgttcaccgg ggtggtgccc atcctggtcg agctggacgg cgacgtaaac ggccacaagt
     1021 tcagcgtgtc cggcgagggc gagggcgatg ccacctacgg caagctgacc ctgaagttca
     1081 tctgcaccac cggcaagctg cccgtgccct ggcccaccct cgtgaccacc ctgacctacg
     1141 gcgtgcagtg cttcagccgc taccccgacc acatgaagca gcacgacttc ttcaagtccg
     1201 ccatgcccga aggctacgtc caggagcgca ccatcttctt caaggacgac ggcaactaca
     1261 agacccgcgc cgaggtgaag ttcgagggcg acaccctggt gaaccgcatc gagctgaagg
     1321 gcatcgactt caaggaggac ggcaacatcc tggggcacaa gctggagtac aactacaaca
     1381 gccacaacgt ctatatcatg gccgacaagc agaagaacgg catcaaggtg aacttcaaga
```

```
1441 tccgccacaa catcgaggac ggcagcgtgc agctcgccga ccactaccag cagaacaccc
1501 ccatcggcga cggccccgtg ctgctgcccg acaaccacta cctgagcacc cagtccgccc
1561 tgagcaaaga ccccaacgag aagcgcgatc acatggtcct gctggagttc gtgaccgccg
1621 ccgggatcac tctcggcatg gacgagctgt acaagtaaag cgatcatcga ctaattcgag
1681 ctcggtaccc ggggatcctc tagagtcgac ctgcaggcat gcaagcttca gaagcgtgct
1741 atcgaactca accagggacg tgcggcacaa atgggcatcc ttgctctcat ggtgcacgaa
1801 cagttgggag tctctatcct tccttaaaaa tttaattttc attagttgca gtcactccgc
1861 tttggtttca cagtcaggaa taacactagc tcgtcttcac catggatgcc aatctcgcct
1921 attcatggtg tataaaagtt caacatccaa agctagaact tttggaaaga gaaagaatat
1981 ccgaataggg cacggcgtgc cgtattgttg gagtggacta gcagaaagtg aggaaggcac
2041 aggatgagtt ttctcgagat actagtacat accttcagcg tcgtcttcac tgtcacagtc
2101 aactgacagt aatcgttgat ccggagagat tcaaaattca atctgtttgg acctggataa
2161 gacacaagag cgacatcctg acatgaacgc cgtaaacagc aaatcctggt tgaacacgta
2221 tccttttggg ggcctcccgc tacgacgctc gctccagctg ggcttccttt actatacaca
2281 gcgcgcatat ttcacggttg ccagatgtca agatcaagat atgatgtttc actataagac
2341 agggtcgtca tggttcctgc tgctgtccgc atccattacc acaaccctca cgacgacaac
2401 gatcacaaca acccacgcct ttgccccgca cacgcgctta tccgtgcccc acggtgcttc
2461 ccgactcgtc atgaaggatt tccccaaacc caatttggaa gatacggaca attaccgttt
2521 gtatcgtgat ttgtcgcact ccttctccac gacactcaag gcaccgtcgc cagaatcacg
2581 caagaaggta gccatcattg gcggtggctt gtccggtctc gcctgtgcca agtacctcgc
2641 cgacgccgga caccaacccg tcgtctacga agcacgggac gtcctcggcg gcaaggtctc
2701 cgcttggcaa gatgccgacg gagactggat cgaaaccgga ctacacattt tctttggtgc
2761 ctacccgaat atgatgaatc tctttgccga acttgatatt cacgatcgtc tccagtggaa
2821 ggtgcacaaa atgatctttg ccatgcagga actacccgga gaattcacaa cctttgattt
2881 cattcccggc atacccgcac ccttcaattt cggactcgct attctcatga accagaaaat
2941 gctcactttg ccggagaaaa tacagacggc tcccccgcta ctgcccatgc tcgtacgcgg
3001 acaagacttt atcgacgaac aggacgaact ctccgtcctc gactttatgc gcaagtacgg
3061 gatgcccgaa cgcatcaacg aagaagtctt tatcagcatg gccaaggcac tcgatttcat
3121 tgatcccgac aaattgagta tgacggtcgt tttgaccgcc atgaatcgct tttgaacgga
3181 agacaacgcc ctccaaatgg cctttctcga cggcaaccag cccgaccggc tctgtgcacc
3241 catggtcgaa cacattcaag cccgtggcgg tcaagtcaac ctcaattccc ccgtgcaaga
3301 aatcgtcacc cgcgaagacg gtagtgtcga ttacctcctg atgcggtccg gagaaaaggt
3361 cgttgccgac gaatacgtgt ccgccatgcc cgtggatatt gtcaaacgca tgttgcccga
3421 aaagtggcaa acaatgccct actttcgtca attcgacgaa ctcgaaggca ttcccgtcat
3481 taacctccac atgtggttcg atcgcaaact caaagccgtc gatcacttgt gcttttcccg
3541 ctcgccgctg ctgtccgttt acgccgacat gagcgtcacc tgcaaggaat actacgacga
3601 atccgcttcc atgctcgagc tcgtctttgc cccgtgcagt ccactcgccg gtggcaacgt
3661 gaattggatt gccaagacgg acgaagaaat catcgacgcg accatgggcg aactggcccg
3721 actctttccc accgaaatcg ccgctgatcc cacctggccg gccaccaaga accagggccc
3781 caacggtgag gccaaacttc gcaaatacgc cgtggtcaag gtgccccgct cggtctacgc
3841 cgccattccc ggccgcaaca agtaccgccc gagtcaaacc actccaattg acaatttcac
3901 gctggccggg gactggacgt cacaaaagtt tctgggatcc atggaaggcg ccgtcctcgg
3961 gggcaagctc gccgccgaag tattggcccg caaggcggcc aacctacccg cacccgaact
4021 cgccaacaaa cccgtgcggg atgaaattgt gcaaaaggcg caaacacacg aagcgcgtcc
4081 accggccgga gtcaagggac aaggtgcgat tgcctttggc ggtggcgccg tgctcgggac
4141 ggaaaacaaa gccttgcttc gggacgttga tcctagtcaa ttcgtggaag cctagatctt
4201 taccttcctt aaaaatttaa ttttcattag ttgcagtcac tccgctttgg tttcacagtc
4261 aggaataaca ctagctcgtc ttcaccatgg atgccaatct cgcctattca tggtgtataa
4321 aagttcaaca tccaaagcta gaacttttgg aaagagaaag aatatccgaa tagggcacgg
4381 cgtgccgtat tgttggagtg gactagcaga aagtgaggaa ggcacaggat gagttttctc
4441 gagactagta tcgaggccgg tctccctata gtgagtcgta ttaatttcga taagccaggt
4501 taacctgcat taatgaatcg gccaacgcgc ggggagaggc ggtttgcgta ttgggcgctc
4561 ttccgcttcc tcgctcactg actcgctgcg ctcggtcgtt cggctgcggc gagcggtatc
4621 agctcactca aaggcggtaa tacggttatc cacagaatca ggggataacg caggaaagaa
4681 catgtgagca aaaggccagc aaaaggccag gaaccgtaaa aaggccgcgt tgctggcgtt
4741 tttccatagg ctccgccccc ctgacgagca tcacaaaaat cgacgctcaa gtcagaggtg
4801 gcgaaacccg acaggactat aaagatacca ggcgtttccc cctggaagct ccctcgtgcg
4861 ctctcctgtt ccgaccctgc cgcttaccgg atacctgtcc gcctttctcc cttcgggaag
4921 cgtggcgctt tctcaatgct cacgctgtag gtatctcagt tcggtgtagg tcgttcgctc
4981 caagctgggc tgtgtgcacg aacccccgt tcagcccgac cgctgcgcct tatccggtaa
5041 ctatcgtctt gagtccaacc cggtaagaca cgacttatcg ccactggcag cagccactgg
5101 taacaggatt agcagagcga ggtatgtagg cggtgctaca gagttcttga agtggtggcc
5161 taactacggc tacactagaa ggacagtatt tggtatctgc gctctgctga agccagttac
5221 cttcggaaaa agagttggta gctcttgatc cggcaaacaa accaccgctg gtagcggtgg
5281 tttttttgtt tgcaagcagc agattacgcg cagaaaaaaa ggatctcaag aagatccttt
5341 gatcttttct acggggtctg acgctcagtg gaacgaaaac tcacgttaag ggattttggt
5401 catgagatta tcaaaaagga tcttcaccta gatcctttta aattaaaaat gaagttttaa
5461 atcaatctaa agtatatatg agtaaacttg gtctgacagt taccaatgct taatcagtga
5521 ggcacctatc tcagcgatct gtctatttcg ttcatccata gttgcctgac tccccgtcgt
5581 gtagataact acgatacggg agggcttacc atctggcccc agtgctgcaa tgataccgcg
5641 agacccacgc tcaccggctc cagatttatc agcaataaac cagccagccg gaagggccga
5701 gcgcagaagt ggtcctgcaa ctttatccgc ctccatccag tctattaatt gttgccggga
5761 agctagagta agtagttcgc cagttaatag tttgcgcaac gttgttgcca ttgctacagg
5821 catcgtggtg tcacgctcgt cgtttggtat ggcttcattc agctccggtt cccaacgatc
5881 aaggcgagtt acatgatccc ccatgttgtg caaaaaagcg gttagctcct tcggtcctcc
5941 gatcgttgtc agaagtaagt tggccgcagt gttatcactc atggttatgg cagcactgca
6001 taattctctt actgtcatgc catccgtaag atgcttttct gtgactggtg agtactcaac
6061 caagtcattc tgagaatagt gtatgcggcg accgagttgc tcttgcccgg cgtcaatacg
6121 ggataatacc gcgccacata gcagaacttt aaaagtgctc atcattggaa aacgttcttc
6181 ggggcgaaaa ctctcaagga tcttaccgct gttgagatcc agttcgatgt aacccactcg
6241 tgcacccaac tgatcttcag catcttttac tttcaccagc gtttctgggt gagcaaaaac
6301 aggaaggcaa aatgccgcaa aaaagggaat aagggcgaca cggaaatgtt gaatactcat
6361 actc
//
```

```
LOCUS       Exported                5330 bp ds-DNA     circular SYN 30-OCT-2018
DEFINITION  pBS-PtPDS1-M1 Phaeodactylum tricornutum nuclear transformation vector
conferring resistance to norflurazon
ACCESSION   MK645853
VERSION     .
KEYWORDS    .
SOURCE      synthetic DNA construct
  ORGANISM  synthetic DNA construct
REFERENCE   1  (bases 1 to 5330)
  AUTHORS   Yogesh Taparia, Aliza Zarka, Stefan Leu, Raz Zarivach, Sammy
            Boussiba, Inna Khozin-Goldberg
  TITLE     A novel endogenous selection marker for the diatom Phaeodactylum
            tricornutum based on a unique mutation in phytoene desaturase 1
            confers resistance to norflurazon
  JOURNAL   Scientific Reports
FEATURES             Location/Qualifiers
     source          1..5330
                     /organism="synthetic DNA construct"
                     /mol_type="other DNA"
     misc_feature    653..725
                     /label=MCS
     promoter        731..976
                     /label=fcpB-Promoter
                     /label=fcpB-P
     CDS             977..2851
                     /label=PtPDS1-M1
                     /label=PtPDS1
     terminator      2852..3093
                     /label=fcpA terminator
     rep_origin      complement(3583..4171)
                     /direction=LEFT
                     /label=ori
     misc_feature    complement(4342..5307)
                     /label=AmpR
ORIGIN
        1 ctaaattgta agcgttaata ttttgttaaa attcgcgtta aatttttgtt aaatcagctc
       61 atttttaac caataggccg aaatcggcaa aatcccttat aaatcaaaag aatagaccga
      121 gatagggttg agtgttgttc cagtttggaa caagagtcca ctattaaaga acgtggactc
      181 caacgtcaaa gggcgaaaaa ccgtctatca gggcgatggc ccactacgtg aaccatcacc
      241 ctaatcaagt tttttggggt cgaggtgccg taaagcacta aatcggaacc ctaaagggag
      301 cccccgattt agagcttgac ggggaaagcc ggcgaacgtg gcgagaaagg aagggaagaa
      361 agcgaaagga gcgggcgcta gggcgctggc aagtgtagcg gtcacgctgc gcgtaaccac
      421 cacacccgcc gcgcttaatg cgccgctaca gggcgcgtcc cattcgccat tcaggctgcg
      481 caactgttgg gaagggcgat cggtgcgggc ctcttcgcta ttacgccagc tggcgaaagg
      541 gggatgtgct gcaaggcgat taagttgggt aacgccaggg ttttcccagt cacgacgttg
      601 taaaacgacg gccagtgagc gcgcgtaata cgactcacta tagggcgaat tgggtaccgg
      661 gcccccccctc gaggtcgacg gtatcgataa gcttgatatc gaattcctgc agcccggggg
      721 atccactagt acataccttc agcgtcgtct tcactgtcac agtcaactga cagtaatcgt
      781 tgatccggag agattcaaaa ttcaatctgt ttggacctgg ataagacaca agagcgacat
      841 cctgacatga acgccgtaaa cagcaaatcc tggttgaaca cgtatccttt tgggggcctc
      901 ccgctacgac gctcgctcca gctggggctt ccttactata cacagcgcgc atatttcacg
      961 gttgccagat gtcaagatga tgtttcacta taagacaggg tcgtcatggt tcctgctgct
     1021 gtccgcatcc attaccacaa ccctcacgac gacaacgatc acaacaaccc acgcctttgc
     1081 cccgcacacg cgcttatccg tgccccacgg tgcttcccga ctcgtcatga aggatttccc
     1141 caaacccaat ttggaagata cggacaatta ccgtttttat cgtgatttgt cgcactcctt
     1201 ctccacgaca ctcaaggcac cgtcgccaga atcacgcaag aaggtagcca tcattggcgg
     1261 tggcttgtcc ggtctcgcct gtgccaagta cctcgccgac gccggacacc aacccgtcgt
     1321 ctacgaagca cgggacgtcc tcggcggcaa ggtctccgct tggcaagatg ccgacggaga
     1381 ctggatcgaa accggactac acattttctt tggtgcctac ccgaatatga tgaatctctt
     1441 tgccgaactt gatattcacg atcgtctcca gtggaaggtg cacaaaatga tctttgccat
     1501 gcaggaacta cccggagaat tcacaacctt tgatttcatt cccggcatac ccgcaccctt
     1561 caatttcgga ctcgctattc tcatgaacca gaaaatgctc actttgccgg agaaaataca
```

1621 gacggctccc ccgctactgc ccatgctcgt acgcggacaa gactttatcg acgaacagga
1681 cgaactctcc gtcctcgact ttatgcgcaa gtacgggatg cccgaacgca tcaacgaaga
1741 agtctttatc agcatggcca aggcactcga tttcattgat cccgacaaat tgagtatgac
1801 ggtcgttttg accgccatga atcgcttttt gaacgaagac aacgccctcc aaatggcctt
1861 tctcgacggc aatcagcccg accggctctg tgcacccatg gtcgaacaca ttcaagcccg
1921 tggcggtcaa gtcaacctca attcccccgt gcaagaaatc gtcacccgcg aagacggtag
1981 tgtcgattac ctcctgatgc ggtccggaga aaaggtcgtt gccgacgaat acgtgtccgc
2041 catgcccgtg gatattgtca aacgcatgtt gcccgaaaag tggcaaacaa tgccctactt
2101 tcgtcaattc gacgaactcg aaggcattcc cgtcattaac ctccacatgt ggttcgatcg
2161 caaactcaaa gccgtcgatc acttgtgctt ttcccgctcg ccgctgctgt ccgtttacgc
2221 cgacatgagc gtcacctgca aggaatacta cgacgaatcc gcttccatgc tcgagctcgt
2281 ctttgccccg tgcagtcctc tcgccggtgg taacgtgaat tggattgcca agacggacga
2341 agaaatcatc gacgcgacca tgggcgaact ggcccgactc ttccccaccg aaatcgccgc
2401 tgatcccacc tggccggcca ccaagaacca gggccccaac ggtgaggcca aacttcgcaa
2461 atacgccgtg gtcaaggtgc cccgctcggt ctacgccgcc attcccggcc gcaacaagta
2521 ccgcccgagt caaaccactc caattgacaa tttcacgctg gccgggggact ggacgtcaca
2581 aaagtttctg ggatccatgg aaggcgccgt cctcgggggc aagctcgccg ccgaagtatt
2641 ggcccgcaag gcggccaacc tacccgcacc cgaactcgcc aacaaacccg tgcgggatga
2701 aattgtgcaa aaggcgcaaa cgcacgtggc gcgtccaccg gccggagtca agggacaagg
2761 tgcgattgcc tttggcggtg gcgccgtgct cgggacggaa aacaaagcct tgcttcggga
2821 cgttgatcct agtcaattcg tggaagccta gaccttcctt aaaaatttaa ttttcattag
2881 ttgcagtcac tccgctttgg tttcacagtc aggaataaca ctagctcgtc ttcaccatgg
2941 atgccaatct cgcctattca tggtgtataa aagttcaaca tccaaagcta gaacttttgg
3001 aaagagaaag aatatccgaa tagggcacgg cgtgccgtat tgttggagtg gactagcaga
3061 aagtgaggaa ggcacaggat gagttttctc gagactagtt ctagagcggc cgccaccgcg
3121 gtggagctcc agcttttgtt ccctttagtg agggttaatt gcgcgcttgg cgtaatcatg
3181 gtcatagctg tttcctgtgt gaaattgtta tccgctcaca attccacaca acatacgagc
3241 cggaagcata aagtgtaaag cctggggtgc ctaatgagtg agctaactca cattaattgc
3301 gttgcgctca ctgcccgctt tccagtcggg aaacctgtcg tgccagctgc attaatgaat
3361 cggccaacgc gcggggagag gcggtttgcg tattgggcgc tcttccgctt cctcgctcac
3421 tgactcgctg cgctcggtcg ttcggctgcg gcgagcggta tcagctcact caaaggcggt
3481 aatacggtta tccacagaat caggggataa cgcaggaaag aacatgtgag caaaaggcca
3541 gcaaaaggcc aggaaccgta aaaaggccgc gttgctggcg tttttccata ggctccgccc
3601 ccctgacgag catcacaaaa atcgacgctc aagtcagagg tggcgaaacc cgacaggact
3661 ataaagatac caggcgtttc cccctggaag ctccctcgtg cgctctcctg ttccgaccct
3721 gccgcttacc ggatacctgt ccgcctttct cccttcggga agcgtggcgc tttctcatag
3781 ctcacgctgt aggtatctca gttcggtgta ggtcgttcgc tccaagctgg gctgtgtgca
3841 cgaacccccc gttcagcccg accgctgcgc cttatccggt aactatcgtc ttgagtccaa
3901 cccggtaaga cacgacttat cgccactggc agcagccact ggtaacagga ttagcagagc
3961 gaggtatgta ggcggtgcta cagagttctt gaagtggtgg cctaactacg gctacactag
4021 aaggacagta tttggtatct gcgctctgct gaagccagtt accttcggaa aaagagttgg
4081 tagctcttga tccggcaaac aaaccaccgc tggtagcggt ggtttttttg tttgcaagca
4141 gcagattacg cgcagaaaaa aaggatctca agaagatcct ttgatctttt ctacggggtc
4201 tgacgctcag tggaacgaaa actcacgtta agggattttg gtcatgagat tatcaaaaag
4261 gatcttcacc tagatccttt taaattaaaa atgaagtttt aaatcaatct aaagtatata
4321 tgagtaaact tggtctgaca gttaccaatg cttaatcagt gaggcaccta tctcagcgat
4381 ctgtctattt cgttcatcca tagttgcctg actccccgtc gtgtagataa ctacgatacg
4441 ggagggctta ccatctggcc ccagtgctgc aatgataccg cgagacccac gctcaccggc
4501 tccagattta tcagcaataa accagccagc cggaagggcc gagcgcagaa gtggtcctgc
4561 aactttatcc gcctccatcc agtctattaa ttgttgccgg gaagctagag taagtagttc
4621 gccagttaat agtttgcgca acgttgttgc cattgctaca ggcatcgtgg tgtcacgctc
4681 gtcgtttggt atggcttcat tcagctccgg ttcccaacga tcaaggcgag ttacatgatc
4741 ccccatgttg tgcaaaaaag cggttagctc cttcggtcct ccgatcgttg tcagaagtaa
4801 gttggccgca gtgttatcac tcatggttat ggcagcactg cataattctc ttactgtcat
4861 gccatccgta agatgctttt ctgtgactgg tgagtactca accaagtcat tctgagaata
4921 gtgtatgcgg cgaccgagtt gctcttgccc ggcgtcaata cgggataata ccgcgccaca
4981 tagcagaact ttaaaagtgc tcatcattgg aaaacgttct tcggggcgaa aactctcaag
5041 gatcttaccg ctgttgagat ccagttcgat gtaacccact cgtgcaccca actgatcttc
5101 agcatctttt actttcacca gcgtttctgg gtgagcaaaa acaggaaggc aaaatgccgc
5161 aaaaaaggga ataagggcga cacggaaatg ttgaatactc atactcttcc tttttcaata
5221 ttattgaagc atttatcagg gttattgtct catgagcgga tacatatttg aatgtattta
5281 gaaaaataaa caaatagggg ttccgcgcac atttccccga aaagtgccac//
